# Self-assembled peptide-substance P hydrogels alleviate inflammation and ameliorate the cartilage regeneration in knee osteoarthritis

**DOI:** 10.1186/s40824-023-00387-6

**Published:** 2023-05-04

**Authors:** Sang Jun Kim, Ji Eun Kim, Goeun Choe, Da Hyun Song, Sun Jeong Kim, Tae Hee Kim, Jin Yoo, Soo Hyun Kim, Youngmee Jung

**Affiliations:** 1 Department of Physical and Rehabilitation Medicine, Seoul Jun Rehabilitation Clinic and Research Center, Seoul, Republic of Korea; 2 Stem Cell & Regenerative Medicine Institute, Samsung Medical Center, Seoul, Republic of Korea; 3 Center for Biomaterials, Biomedical Research Institute, Korea Institute of Science and Technology (KIST), Seoul, 02792 Republic of Korea; 4 KU-KIST Graduate School of Converging Science and Technology, Korea University, Seoul, Republic of Korea; 5 Stem Cell Institute, ENCell Co. Ltd, Seoul, Republic of Korea; 6 Cell and Gene Therapy Institute, Samsung Medical Center, Seoul, Republic of Korea; 7 School of Electrical and Electronic Engineering, YU-KIST Institute, Yonsei University, Seoul, Republic of Korea

**Keywords:** Self-assembled peptide, Substance P, Mesenchymal stem cells, Osteoarthritis

## Abstract

**Background:**

Self-assembled peptide (SAP)-substance P (SP) hydrogels can be retained in the joint cavity longer than SP alone, and they can alleviate inflammation and ameliorate cartilage regeneration in knee osteoarthritis (OA). We conducted a preclinical study using diverse animal models of OA and an in vitro study using human synoviocytes and patient-derived synovial fluids to demonstrate the effect of SAP-SP complex on the inflammation and cartilage regeneration.

**Methods:**

Surgical induction OA model was prepared with New Zealand white female rabbits and chemical induction, and naturally occurring OA models were prepared using Dunkin Hartely female guinea pigs. The SAP-SP complex or control (SAP, SP, or saline) was injected into the joint cavities in each model. We performed micro-computed tomography (Micro-CT) analysis, histological evaluation, immunofluorescent analysis, and terminal deoxynucleotidyl transferase deoxyuridine triphosphate nick-end labeling (TUNEL) assay and analyzed the recruitment of intrinsic mesenchymal stem cells (MSCs), macrophage activity, and inflammatory cytokine in each OA model. Human synoviocytes were cultured in synovial fluid extracted from human OA knee joints injected with SAP-SP complexes or other controls. Proliferative capacity and inflammatory cytokine levels were analyzed.

**Results:**

Alleviation of inflammation, inhibition of apoptosis, and enhancement of intrinsic MSCs have been established in the SAP-SP group in diverse animal models. Furthermore, the inflammatory effects on human samples were examined in synoviocytes and synovial fluid from patients with OA. In this study, we observed that SAP-SP showed anti-inflammatory action in OA conditions and increased cartilage regeneration by recruiting intrinsic MSCs, inhibiting progression of OA.

**Conclusions:**

These therapeutic effects have been validated in diverse OA models, including rabbits, Dunkin Hartley guinea pigs, and human synoviocytes. Therefore, we propose that SAP-SP may be an effective injectable therapeutic agent for treating OA.

**Graphical Abstract:**

In this manuscript, we report a preclinical study of novel self-assembled peptide (SAP)-substance P (SP) hydrogels with diverse animal models and human synoviocytes and it displays anti-inflammatory effects, apoptosis inhibition, intrinsic mesenchymal stem cells recruitments and cartilage regeneration

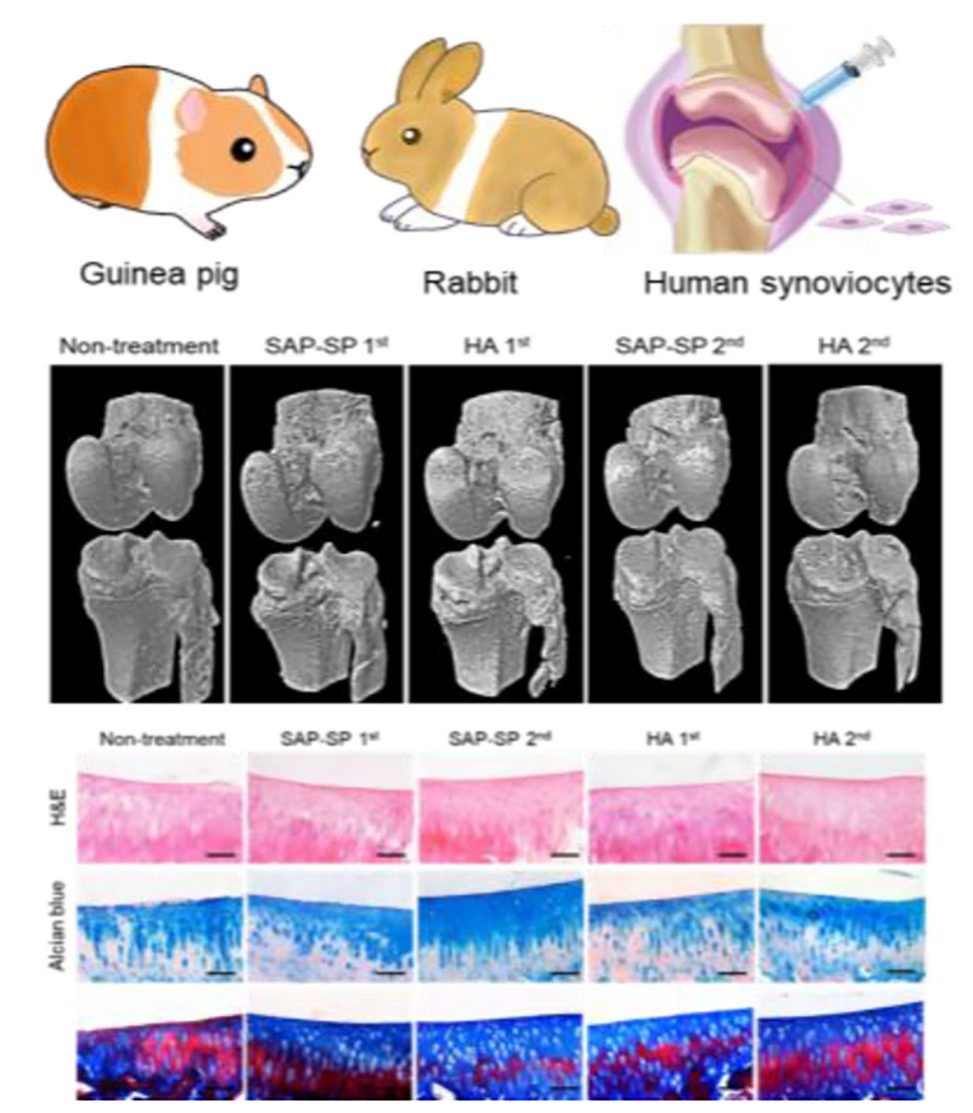

**Supplementary Information:**

The online version contains supplementary material available at 10.1186/s40824-023-00387-6.

## Introduction

Osteoarthritis (OA) is a degenerative disease characterized by articular cartilage loss, subchondral bone sclerosis, and synovial inflammation [1]. Although there have been many therapeutic tools including oral medication and intraarticular injection for OA, most treatments have focused on symptomatic relief rather than a reversal of regenerative changes [2].

Recently, several trials have been conducted for developing new drugs that enhance regeneration and inhibit the progression of degenerative changes in OA. Strontium ranelate [3], discoidin domain receptor blocker [4], avocado [5], vitamin D [6], and vitamin E [7] are examples of these investigative therapies. None of them showed clear effects, possibly because the exact pathophysiology of OA is still under investigation [8]. Inflammation related to cytokines and chemokines is considered a major pathophysiological mechanism of OA. Cartilage destruction and osteoarthritic changes occur as secondary processes caused by inflammation [9–11].

Substance P (SP) is an intrinsic substance related to the immune response and is involved in many biological processes, including nociception and inflammation [12, 13]. SP is localized throughout the subchondral bone, contributing to OA pain, and it serves various biological functions such as neurokinin-1 receptor (NK1R) activation, stem cell recruitment, and tissue regeneration [14–19]. In our previous study, intraarticular injection of SP combined with an adequate scaffold delayed the progression of OA and improved the symptoms of OA [20]. Considering that the major pathologic processes in OA are synovial inflammation and cartilage degradation, the therapeutic potentials of SP on OA are explainable, and intraarticular injection of SP complexes can be considered as a new therapeutic agent for OA. However, because of low molecular weight of SP, its residence time in the joint is short. Although no studies have been conducted on the residence time of SP in the joint cavity, the residence time of SP in the intrathecal space was shown to be 4 h [21]. Therefore, a longer residence time is required for the use of SP as a therapeutic agent for OA.

A self-assembled peptide (SAP) is a conjugate of hydrophilic and hydrophobic groups that can self-assemble into aligned nanostructures [22, 23]. SAP has been widely used in the regenerative medical fields because of its ability to support cell attachment, migration, and differentiation and to increase the residence time of implanted cells [24–26]. The hydrogels formed by SAP reside in tissue by forming extracellular matrices, which assist in the function of stem cells by binding to the target receptor [18]. Therefore, we hypothesized that SAP would assist the function of SP by increasing the residence time of SP in the joint cavity. In our previous study, we found that SAP-SP complexes remained in the joint cavity of rats for 6 weeks [27].

Despite promising results in preclinical studies of new therapeutics, the transition to clinical trials often fails. Possible causes of this failure are the lack of an ideal OA animal model, mismatch of pharmacokinetics and pharmacodynamics between animals and humans, and inadequate outcome parameters in animal studies [28, 29]. Although there are several animal models of OA, each has its unique strengths and weaknesses with respect to its adaptation to the clinical setting [30–32]. Therefore, individual animal models of OA are insufficient for generalizing preclinical results to clinical settings. Considering that human primary OA progresses slowly over a period, the Dunkin Hartley guinea pig is adequate as a spontaneous OA model for evaluating the response of therapeutic agents in slowly progressive OA [33, 34]. However, because human primary OA is aggravated by predisposing factors such as knee joint instability, an animal OA model developed using anterior cruciate ligament (ACL) resection is more adequate [35].

Synoviocytes play a role in OA progression by modulating the expression of inflammatory cytokines [36, 37]. A human synoviocyte in vitro model of OA has been used for demonstrating pharmacological effects [38, 39]; this model has the advantage of simulating human OA environments. In vivo studies using various animal models of OA and in vitro studies using human OA tissues will reduce the gap between preclinical research and clinical trials of new therapeutic agents for OA. We conducted a preclinical study using diverse animal models of OA to demonstrate the effect of SAP-SP complexes on OA progression.

## Materials and methods

### Preparation of SAP-SP complex

For the preparation of SAP and SAP-SP complexes, the peptides KLD12 (Ac-KLDLKLDLKLDL-NH_2_) and KLD12-SP (Ac-KLDLKLDLKLDLGGRPKPQQFFGLM-NH_2_), synthesized by Peptron (Daejeon, Korea), were dissolved in 295 mM sucrose solution, which yielded a 1% (w/v) peptide gel. The peptides were synthesized by Fmoc solid-phase peptide synthesis (SPPS) using ASP48S (Peptron) and purified by reverse-phase high-performance liquid chromatography using a Vydac® Everest® C18 column (250 × 22 mm, 10 μm; Avantor Inc., Radnor, PA, USA). Elution was performed using a water–acetonitrile linear gradient (3–40% (v/v) acetonitrile) containing 0.1% (v/v) trifluoroacetic acid. The molecular weights of the purified peptides were confirmed using Agilent HP1100 series LC/MS (Agilent Technology, Santa Calara, CA, USA). For the preparation of SAP, the 1% KLD12 peptide gel was diluted with phosphate-buffered saline (PBS) and sonicated for 30 min to form a 0.5% KLD12 peptide hydrogel. To prepare the SAP-SP complex, each peptide gel containing 0.5% KLD12 and 1% KLD12-SP was mixed at a 70:1 (v/v) concentration.

### OA animal model

The procedures used in this study were approved by the Institutional Animal Care and Use Committee of the Korea Institute of Science and Technology and Samsung Medical Center, in accordance with the recommendations for handling laboratory animals for biomedical research (approval No. 2014–06068, 201,602,125,001). Human OA develops from diverse conditions, such as trauma, inflammation, and senile changes, which are investigated in diverse animal OA models, including surgical, chemical induction, and naturally occurring conditions. To minimize individual variance, OA models were prepared in both rabbit hindlimbs, and interventions were only performed in the left hindlimbs to confirm OA changes in the right hindlimbs and acquire the differential ratio between sides. All animals were randomly allocated to experimental groups.

#### Surgical OA model

For the surgical induction of OA, 20 New Zealand white female rabbits (weight, 3–3.2 kg; age, 19 weeks) were anesthetized with an intramuscular injection of 35 mg/kg ketamine (Ketamine 50®, Yuhan Corp, Seoul, Korea) and 5 mg/kg xylazine (Rompun®, Bayer Vital GmbH, Leverkusen, Germany) and maintained with 2% isoflurane (Ifran®, Hana Pharm, Hwaseong, Korea). The anterior portions of both hind limbs were shaved with an electric clipper and cleansed with povidone-iodine. The skin around the knee cap was incised vertically along the medial border of the knee cap. With the knee joint flexed, the patella was pushed laterally to expose the ACL. The anterior cruciate and medial collateral ligaments were then cut, and the medial meniscus was completely extracted with surgical scissors and forceps without injury to the cartilage of the femur or tibia. The patella was then replaced, and the knee cap, fascia, and skin were closed with 4 − 0 polydioxanone sutures. A 5-day course of 5 mg/kg enrofloxacin (Baytril®; Bayer, Milan, Italy) and 40 mg/kg ketoprofen (Ketoprofen®; Unibiotech, Yesan, Korea) was administered for preventing postoperative infection. After the surgery, each rabbit was placed in a cage with free movement for 3 weeks.

#### Chemical induction OA model

An monosodium iodoacetate (MIA) injection model was used as the chemical-induction OA model. For the induction of MIA-induced arthritis, 16 Dunkin Hartley female guinea pigs 15 weeks old (779.4-854.3 g) were used. The guinea pigs were allowed to acclimate to the facility for seven days. The MIA was dissolved in physiological saline (100 µg/µL) and filtered using a syringe filter. The diluted MIA solution (100 µL) was administered with a 26-gauge needle into the joint cavity of the right knee of the guinea pig [40].

#### Naturally occurring OA model

For the induction of naturally occurring OA, Dunkin Hartley female guinea pigs (age, 15 weeks) were used. Guinea pigs were housed for 44 weeks (978.2-1089.5 g) to observe the therapeutic effects of the naturally occurring OA model. This method was used in previous studies [40–42].

### Injection of SAP-SP complex into the joint cavity

In the surgical OA model, 14 rabbits were randomly allocated into 4 groups: the SAP-SP group (n = 3), SAP group (n = 3), SP group (n = 3), and saline group (n = 3) according to the injection materials given. We optimized the dose and injection protocols based on our previous study [27], and when we used different animal species, we adjusted the dosage by body weight at first and readjusted the dosage after injection. Three weeks after the surgical induction of OA, 0.45 mL of each material was injected into the left knee joint cavity. The rabbits were sacrificed at 6 months to investigate changes in OA.

**Fig. 1:**
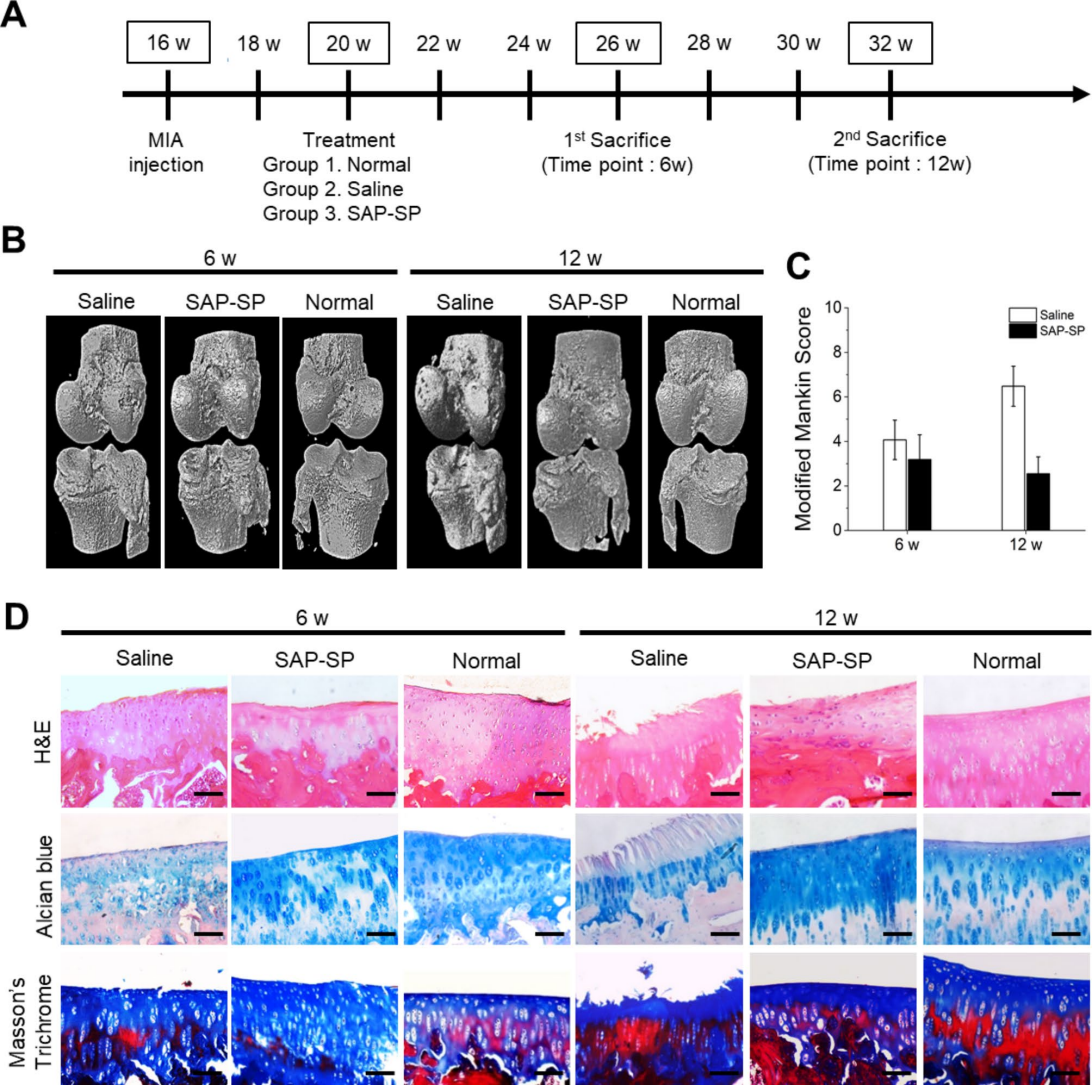
**(A)** Schematic timeline for the treatment for the chemical induction OA models, micro-CT images of guinea pig knee joint of the chemical induction models (n = 8 in each group). **(B)** At 4 weeks post-chemical induction, the knee joints of the guinea pigs were extracted for a micro-CT analysis. **(C)** Modified Mankin Score and **(D)** Histological studies of guinea pig knee joint of chemical induction model at 6 and 12 weeks after chemical induction. The figure showed macroscopic images of hematoxylin and eosin (upper line), alcian blue (middle line), and Masson’s trichrome staining (lower line). Scale bars: 200 μm

In the chemical induction OA model, 24 Dunkin Hartley guinea pigs were randomly allocated into 3 groups; 16 underwent MIA injection, while 8 functioned as untreated controls. The 16 animals that received the injection were divided into the SAP-SP treatment group (n = 8) and saline treatment group (n = 8). At 4 weeks after the MIA injection, after confirming the full development of OA, 200 µL of SAP-SP or saline was injected into the right knee joint cavities of the guinea pigs in those groups; nothing was injected into the right knee joint cavities of the untreated group. Four guinea pigs in each group were sacrificed at 6 and 12 weeks to investigate changes in OA. A schematic timeline of this chemical induction OA model was presented in Fig. 1A.

**Fig. 2:**
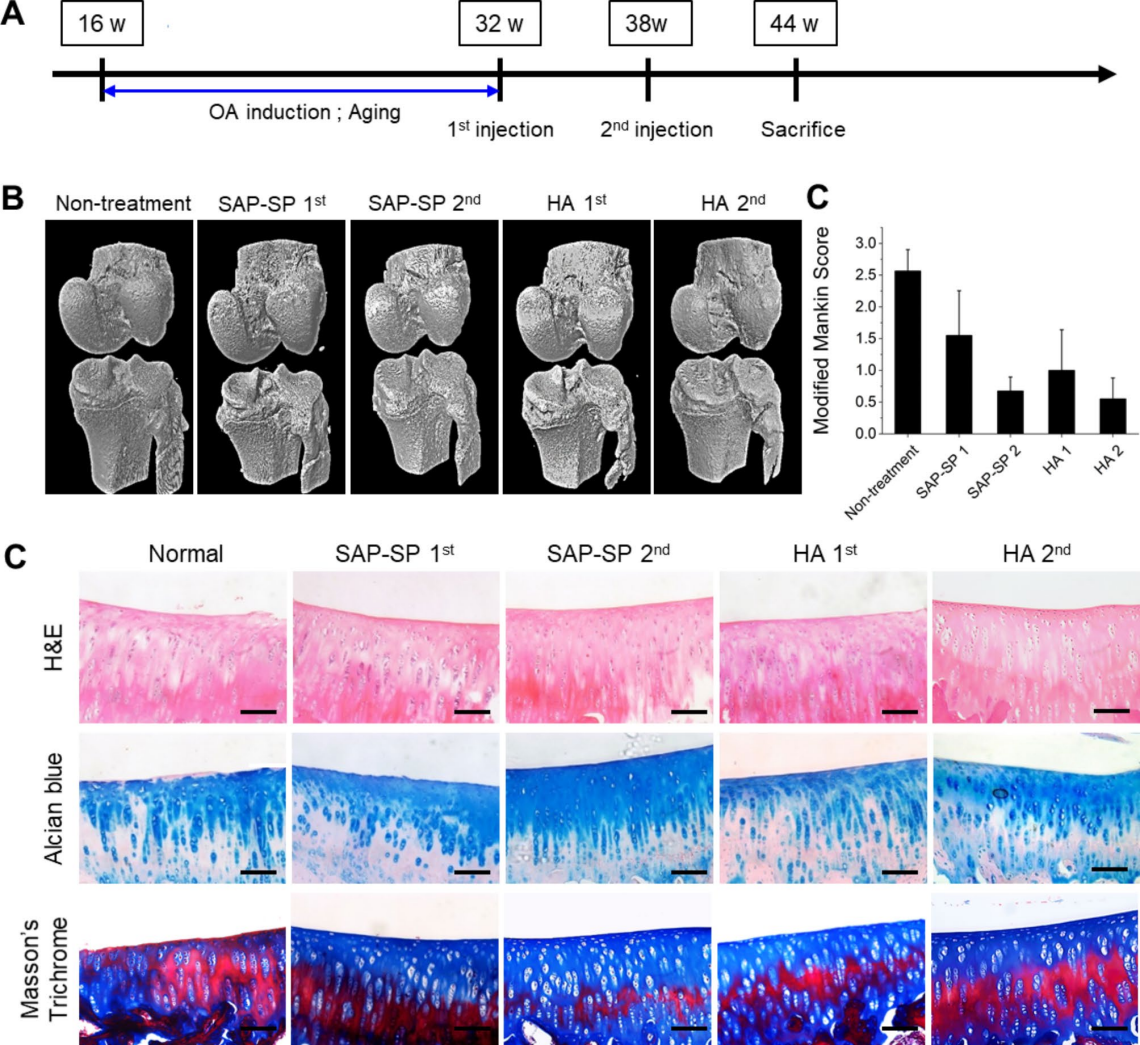
**(A)** Schematic timeline for the treatment for the spontaneous occurring models, Micro-computed tomography images of guinea pig knee joint of the spontaneous occurring models (n = 8 in the non-treatment group, n = 4 in the other groups). **(B)** At 44 weeks, the knee joints of the guinea pigs were extracted for a micro-CT analysis, **(C)** Modified Mankin Score, and **(D)** histological studies of knee joints of guinea pigs. The figure shows macroscopic images of hematoxylin and eosin (upper line), alcian blue (middle line), and Masson’s trichrome staining (lower line). Scale bars: 200 μm

In the naturally occurring OA model, 24 Dunkin Hartley guinea pigs were randomly allocated into five groups: the non-treatment group (naturally occurring OA, n = 8), single SAP-SP injection group (SAP-SP injection at 12 weeks, n = 4), single hyaluronic acid (HA) injection group (HA injection at 12 weeks, n = 4), repeated SAP-SP injection group (one injection every 6 weeks for 12 weeks, n = 4), and repeated HA injection group (one injection every 6 weeks for 12 weeks, n = 4). Each treatment (0.2 mL) was administered to the right knee joint cavity. Hyaluronic acid was obtained from LG Chemistry (Hyruan Plus^®^; 10 mg/mL, 3.0 $\times$ 10^6^ Da, LG Chemistry, Seoul, Korea). Twelve weeks after treatment, the guinea pigs were sacrificed to investigate changes in OA. A schematic timeline of this naturally occurring OA model experiment was presented in Fig. 2A.

### Analysis of animal studies

#### Micro-CT analysis

Both knee joints of all rabbits and guinea pigs were extracted for micro-CT analysis using a Siemens Inveon Micro-PET/CT scanner (Siemens Medical Solutions, Knoxville, USA). All samples were scanned with a 1.5 mm aluminum filter using the following settings: 360° total rotation and 720 rotation steps yielding a 0.5° step size, 70 kV and 400 µA source settings, and 800 ms exposure time per step. The pixels were binned by two, resulting in an effective pixel size or resolution of approximately 20.26 μm.

For each scan, the dataset was reconstructed with a down sample factor of two using the Inveon Acquisition Workplace software package (Siemens Medical Solutions), implementing the modified Feldkamp filtered back projection algorithm (Shepp-Logan filter). The reconstructed images were then imported using the Inveon Research Workplace software (Siemens Medical Solutions) into the accompanying two-dimensional (2D) and three-dimensional (3D) biomedical image analysis software package CT Bone Visualization and Analysis. The knee joint was divided into four compartments: the medial femur (MFC), lateral femur (LFC), medial tibia (MTP), and lateral tibia (LTP), each containing anterior, central, and posterior cylindrical regions of interest (ROIs), providing a total of 14 sample locations per knee joint. Cylindrical ROIs were created with a diameter of approximately 1.8 mm and length of 1.8 mm, giving an ROI volume of approximately 4.6 mm^3^. Bone mineral density (BMD) measurements were calculated by scanning a phantom (same acquisition settings as the femurs) containing rods of known density values and creating a linear calibration curve (R^2^ = 0.9987) relating these densities to their measured Hounsfield (HU) values upon scanning.

Considering the individual differences in BMD, the value of BMD on the left side was converted to a ratio (%) by dividing it by the value of the right-side BMD at which the OA model was constructed; however, no injection was performed.

#### Histological analysis

Specimens from the knee joints of rabbits and guinea pigs were stained for histological analysis. The collected samples were fixed in 10% (v/v) buffered formalin, decalcified with a hydrogen chloride ethylenediaminetetraacetic acid solution, and then embedded in paraffin. The specimens were sectioned in the sagittal plane under the midline at a thickness of 5 µm. To observe collagen and sulfated glycosaminoglycans (GAGs), we performed alcian blue staining. The sections were stained with H&E to identify the nucleus and cytoplasm.

A modified Mankin scoring system was used to evaluate the OA changes. All grades were rated by a blinded researcher. The items of the scoring system were cartilage structure, cartilage cells, alcian blue staining, and tidemark integrity. A detailed description of this system has been reported in a previous article [27].

For immunofluorescence analysis, the cells were stained with goat polyclonal antibody for matrix metalloproteinase-9 (MMP-9) (sc-6841, Santa Cruz Biotechnology, Texas, USA), mouse monoclonal antibody for type II collagen (sc-52658, Santa Cruz Biotechnology), and mouse monoclonal antibody for transforming growth factor beta 1 (TGF-β1; MA1-21595, Invitrogen, Carlsbad, CA, USA) at a 1:100 dilution. Alexa Fluor 488 donkey anti-goat IgG (ab150129, Abcam), Alexa Fluor 594 rabbit anti-mouse IgG (A-11062, Invitrogen), and Alexa Fluor 594 goat anti-mouse IgG (A-11032, Invitrogen) were used as secondary antibodies at a 1:1000 dilution. To reduce the effect of auto-fluorescence in the skeletal tissues, samples were immersed in sudan black B solution (0.1 g Sudan black B in 100 mL EtOH) at 25°C for 30 min. After washing steps with MHB solution (NaCl 80 g, KCl 0.4 g, KH_2_PO_4_ 60 mg, glucose 1 g, Na_2_HPO_4_ 47.9 mg, EGTA 0.2 mM, MES 0.5 mM), nuclei were counterstained with 4’,6-diamidino-2-phenylindole (DAPI; Thermo Fisher Scientific, Waltham, MA, USA). Immunofluorescence staining was performed as previously described [20].

#### TUNEL assay

Apoptosis in the cartilage of the knee joint was detected using the DeadEnd™ fluorometric TUNEL system (G3250, Promega, Madison, WI, USA) according to the manufacturer’s instructions. Stained specimens (n = 3 in each group) were examined using fluorescence microscopy. For quantitative analysis of apoptotic cells, the number of TUNEL + nuclei and total number of nuclei were counted in three different randomized fields (200× magnification). The TUNEL + cell density (%) was expressed as the ratio of TUNEL + nuclei to the total number of nuclei.

#### Recruitment of MSCs

To investigate MSC recruitment by the SAP-SP complex, sections of knee joints were double-stained with antibodies against MSC markers CD29 (sc-6622, goat anti-integrin beta 1 polyclonal antibody, Santa Cruz Biotechnology), and CD90 (ab225, mouse anti-CD90/Thy1 monoclonal antibody, Abcam) at a 1:100 dilution. Alexa Fluor 488 donkey anti-goat IgG and Alexa Fluor 594 rabbit anti-mouse IgG were used as the secondary antibodies at a 1:1000 dilution. The nuclei were counterstained with DAPI. The specimens were observed using fluorescence microscopy (Eclipse TE2000U), and images were taken in four random fields at 200× magnification (n = 4). The stained areas were analyzed as the mean per unit area (mm^2^/mm^2^) using the ImageJ software (NIH, Maryland, USA).

#### Immune response evaluation

To investigate the immune response after injection of various materials such as saline, SP, and SAP-SP, macrophage markers including CD68 (mouse anti-CD68 monoclonal antibody, ab201340, Abcam) and CD206 (goat anti-CD206 polyclonal antibody, sc-34,577, Santa Cruz Biotechnology) were used for immunofluorescence analysis at a 1:100 dilution. Alexa Fluor 488 donkey anti-goat IgG and Alexa Fluor 594 rabbit anti-mouse IgG were used as the secondary antibodies at a 1:1000 dilution. DAPI was used to counterstain nuclei. The macrophage activity of the specimens was examined according to protocols used in a previous study [27].

#### In vivo disappearance analysis

SAP and SAP-SP were tagged with molecular tags via chemical bonding with biotin and tetramethylrhodamine (TAMRA), respectively (Peptron). To investigate the in vivo disappearance, a peptide gel was prepared in the same manner as in the previous method using biotinylated SAP and TAMRA-tagged SAP-SP. Unbound peptides were removed by extensive washing. Ten rats were surgically induced to develop OA. The prepared biotinylated SAP (0.2 mL) was injected 3 weeks after surgery. At 1, 3, and 5 months after the injection, the synovial fluid was washed with PBS using a syringe and extracted with another syringe (as described in the rabbit synovial fluid analysis section). To estimate biotin-labeled incorporation, the synovial fluid containing the labeled biotin was added to a mixture of 4-hydroxyazobenzene-2-carboxylic acid and avidin (Thermo Fisher Scientific). The resulting solution was added to 96-well plates, and the plates were examined using a microplate reader. The absorbance was measured at 500 nm. The disappearance of biotinylated SAP was detected using DyLight 594-conjugated streptavidin (22,842, Thermo Fisher Scientific) staining. In each group, four rabbits were sacrificed at each time point. The stained samples were examined using fluorescence microscopy.

#### Rabbit synovial fluid analysis

The synovial fluid was aspirated from the left knee joint of rabbits using a syringe with a 23 G needle 18 weeks after the SAP-SP complex injection. To quantify and identify the cytokines secreted from the synovial fluid of the knee joints during the treatment process, 250 µL of synovial fluid from each group was prepared. Cytokine evaluation was performed according to the manufacturer’s instructions using the Proteome Profiler™ Human XL Cytokine Array Kit (ARY022B, R&D Systems, Minneapolis, MN, USA). This kit detects chitinase 3-like 1 (CHI3L1), interleukin-1α (IL-1α), interleukin-17 A (IL-17 A), IL-19, interferon-γ (IFN-γ), pentraxin-3 (PTX-3), and stromal cell-derived factor-1α (SDF-1α). The spot pixel density profiles were analyzed using the LAS 3000 imaging system (Fujifilm, Tokyo, Japan) and the ImageJ software.

#### Quantitative real-time polymerase chain reaction

For quantitative gene expression in the articular cartilage of the knee joints, rabbits and guinea pigs were sacrificed. The articular cartilage specimens were closely incised using fine scissors. RNA was extracted from the minced articular cartilage tissues according to the manufacturer’s instructions using the RNeasy Mini Kit (74106, QIAGEN, Hilden, Germany). RNA from each sample was reverse-transcribed into cDNA using an Omniscript system kit (QIAGEN). The synthesized cDNA was examined using an Applied Biosystems 7500 real-time PCR (RT-PCR) system with Power SYBR® Green PCR Master Mix (Applied Biosystems, Massachusetts, USA). Glyceraldehyde-3-phosphate dehydrogenase (GAPDH) primers were used for normalizing each sample. Interleukin-1β (IL-1β) is associated with a pro-inflammatory response and caspase-3 is an indicator of apoptosis. As chondrogenic markers, aggrecan (ACAN) and TGF-β1 were also examined by RT-PCR. Primers were produced from Bioneer (Daejeon, Korea). The primer sequences for the individual genes used were as follows [43]: GAPDH (Rabbit; FW: 5’-TGA CGA CAT CAA GAA GGT GGT G-3’, RV: 5’-GAA GGT GGA GGA GTG GGT GTC-3’), ACAN (Rabbit; FW: 5’-GCT ACG GAG ACA AGG ATG AGT TC-3’, RV: 5’-CGT AAA AGA CCT CAC CCT CCA T-3’), Caspase-3 (Rabbit; FW: 5’-GCT GGA CAG TGG CAT CGA GA-3’, RV: 5’-TCC GAA TTT CGC CAG GAA TAG TAA-3’), TGF-β1 (Rabbit; FW: 5’-CAG TGG AAA GAC CCC ACA TCT C-3’, RV: 5’-GAC GCA GGC AGC AAT TAT CC-3’), GAPDH (Guinea pig; FW: 5’-GTA TCG TGG AAG GAC TCA TGA CC-3’, RV: 5’-GTT GAA GTC ACA GGA CAC AAC CT-3’), tumor necrosis factor-α (TNF-α) (Guinea pig; FW: 5’-CCT ACC TGC TTC TCA CCC ATA CC-3’, RV: 5’-TTG ATG GCA GAG AGA AGG TTG A-3’), interleukin-10 (IL-10) (Guinea pig; FW: 5’-GGC ACG AAC ACC AGT CTG A-3’, RV: 5’-TCA CCT GCT CCA CTG CCT TG-3’).

### Analysis of human samples

In this study, we used human synovial fluid to monitor the cell growth of synoviocytes and the change in secreted pro- or anti-inflammatory cytokines when our peptide hydrogel was injected into the synovial fluid of patients with OA. Human synovial fluid samples were obtained from six patients with OA (average age 67.3 ± 7.5 years old) with the approval of the Samsung Medical Center of Korea Institutional Review Board (IRB approval number 2018-01-026). Osteoarthritis in all the patients was primary OA, not secondary OA. X-ray examination showed medial joint line narrowing and bony spurs. The OA severity was Kellgren-Lawrence grade III. Synoviocytes (human fibroblast-like synoviocyte cell line; passage 2) were purchased from Cell Applications Inc (San Diego, CA, USA). The cells were cultured in flasks in synoviocyte growth medium (Cell Applications) at 37 °C under standard cell culture conditions (humidified atmosphere of 5% CO_2_). After passaging four times, the cells (1 × 10^4^ cells/well) were seeded in 24-well plates for one day. Human synovial fluid (50 µL), cell culture medium (minimum essential medium with 10% fetal bovine serum and 1% penicillin and streptomycin; 750 µL), and treatment materials (200 µL) were added to each well for confirming the effects of the therapeutics on the human samples. The six experimental groups were as follows: synovial fluid (SF) + medium + SAP, SF + medium + soluble SP, SF + medium + SAP-SP, SF + medium + HA, SF + medium (non-treatment group), and medium (no synovial fluid). All sample groups were cultured for 14 days in a humidified incubator at 37 °C under 5% CO_2_, and these experiments were repeated four times for ensuring consistency in the results. The growth of the synoviocytes was examined using a cell counting kit-8 (WST assay, Dojindo Laboratories, Kumamoto, Japan) at 1, 3, 7, and 14 days. Briefly, the supernatants were aspirated, and 450 µL of culture medium and 50 µL of cell counting solution were added to each well. After incubation at 37 °C under 5% CO_2_ for 4 h, the resulting solutions (100 µL per well) were added to 96-well plates, and the plates were read using a microplate reader. The absorbance was measured at 450 nm. Furthermore, to identify cytokine changes in synovial fluid by treatment injection, the supernatants of each well were collected every 2 days and immediately stored at -80 ℃. A Luminex assay was performed according to the manufacturer’s instructions using high-sensitivity human IL-1β, TNF-α, and interleukin-4 (IL-4) Luminex assay kits (R&D Systems, Minneapolis, MN, USA).

### Statistical analysis

Statistical analysis was performed using SPSS 20.0 (IBM, Armonk, NY, USA) for evaluating the correlation between the various treatments and experimental results. For the data from the synovial fluid analysis, repeated measures ANOVA was conducted for determining whether there were significant differences between the treatment groups and different time points. For other experiments, Kruskal–Wallis and post-hoc Mann–Whitney U tests with Bonferroni correction were used for determining the differences between the groups. A *p*-value less than 0.05 was considered statistically significant.

## Results

### The effects of SAP-SP on OA in the surgical OA models

**Fig. 3:**
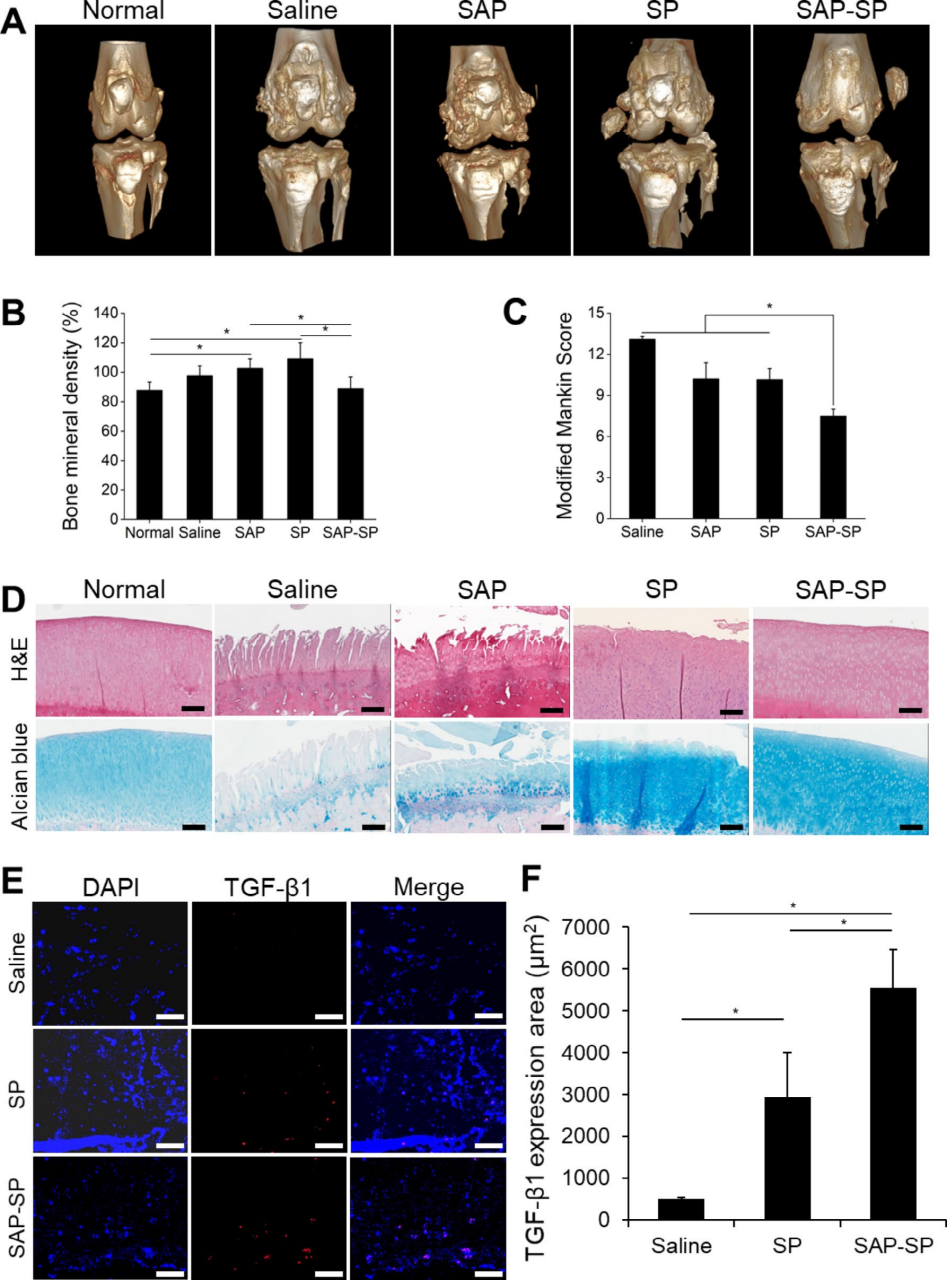
**(A)** Micro-CT images of rabbit knee joints from the surgical induction model at 6 months post-surgical induction. **(B)** Bone mineral density (BMD) was calculated by scanning a phantom from the micro-CT results (n = 3 in each group, (*, *p* < 0.05 by Kruskal–Wallis and post-hoc Mann–Whitney U tests with Bonferroni correction). **(C)** Evaluation of the Modified Mankin score according to cartilage structure, cartilage cells, alcian blue staining, and tidemark integrity (*, *p* < 0.05). **(D)** Histological studies of the rabbit knee joint surgical induction model at 6 months after surgical induction. The figure shows macroscopic images of H&E (upper line) and alcian blue (lower line) staining. Scale bars: 200 μm. **(E)** The expression of TGF-β1 in the subchondral bone in the immunofluorescent-stained images and **(F)** the quantified TGF-β1 expression area for each group (µm^2^) (*, *p* < 0.05). Scale bars: 100 μm

After euthanizing the rabbits and extracting the joint structures six months after the injection, 3D images of the knee joints were acquired using micro-CT (Fig. 3A). The articular surface of the knee joints in the saline and SAP groups was rough, and subchondral bone sclerosis was observed. The articular surface in the SP group was relatively smooth compared with that in the control group; however, subchondral bone sclerosis was similar. The articular surface in the SAP-SP group was smooth, and subchondral bone sclerosis was less severe than that in the other groups. The saline group exhibited a BMD of 99.4%, whereas the SAP and SP groups exhibited BMDs of 106.1% and 109.7%, respectively. However, in the SAP-SP group, the BMD decreased to 87.3% (Fig. 3B).

To assess the changes in the subchondral bone, hematoxylin and eosin (H&E), and alcian blue staining were performed (Fig. 3D). Many clefts deep in the bone tidemark and hypocellularity were found in the saline and SAP groups. GAG staining was low in the saline and SAP groups. The clefts were present only at the depth of the transitional zone, and their numbers decreased in the SP group. In addition, the number of chondrocytes and level of GAG staining were higher in the SAP group than in the control group. However, the SP group showed an irregular articular surface and pyknosis of chondrocytes, indicating incomplete recovery of the articular joints. In contrast, the articular surfaces of the SAP-SP group recovered to the level observed for the normal control. The articular surface was smooth, and the shape and number of chondrocytes and GAG content were similar to that in the normal control. The modified Mankin score was 13.1 ± 0.9 in the saline group, 10.3 ± 3.4 in the SAP group, 8.9 ± 2.5 in the SP group, and 7.4 ± 1.4 in the SAP-SP group (Fig. 3C).

To confirm the anti-inflammatory effects of SAP-SP for treating OA, we performed immunofluorescence staining showing the expression of TGF-β1 in the subchondral bone (Fig. 3E and F). The TGF-β family is also known to stimulate chondrogenesis in mesenchymal cells and inhibit hypertrophic differentiation of chondrocytes [44]. TGF-β1 expression was slightly enhanced in the SP group and was greatly enhanced in the SAP-SP group. In addition, to analyze cartilage regeneration resulting from SAP-SP injection at the genetic level, the gene expression of ACAN, caspase 3, and TGF-β1 was estimated using qRT-PCR (Fig. S1). ACAN and TGF-β1 expression were slightly elevated in the SP group (0.66 ± 0.02 and 2.67 ± 1.11, respectively) and highly elevated in the SAP-SP group (1.64 ± 0.38 and 4.6 ± 0.58) compared to that in the saline group (0.14 ± 0.02 and 0.13 ± 0.07, *p* = 0.027). In addition, the expression of caspase-3 was lower in the SP group than in the saline group. However, although its expression was lower in the SAP-SP group, the difference was not statistically significant (*p* = 0.051).

**Fig. 4:**
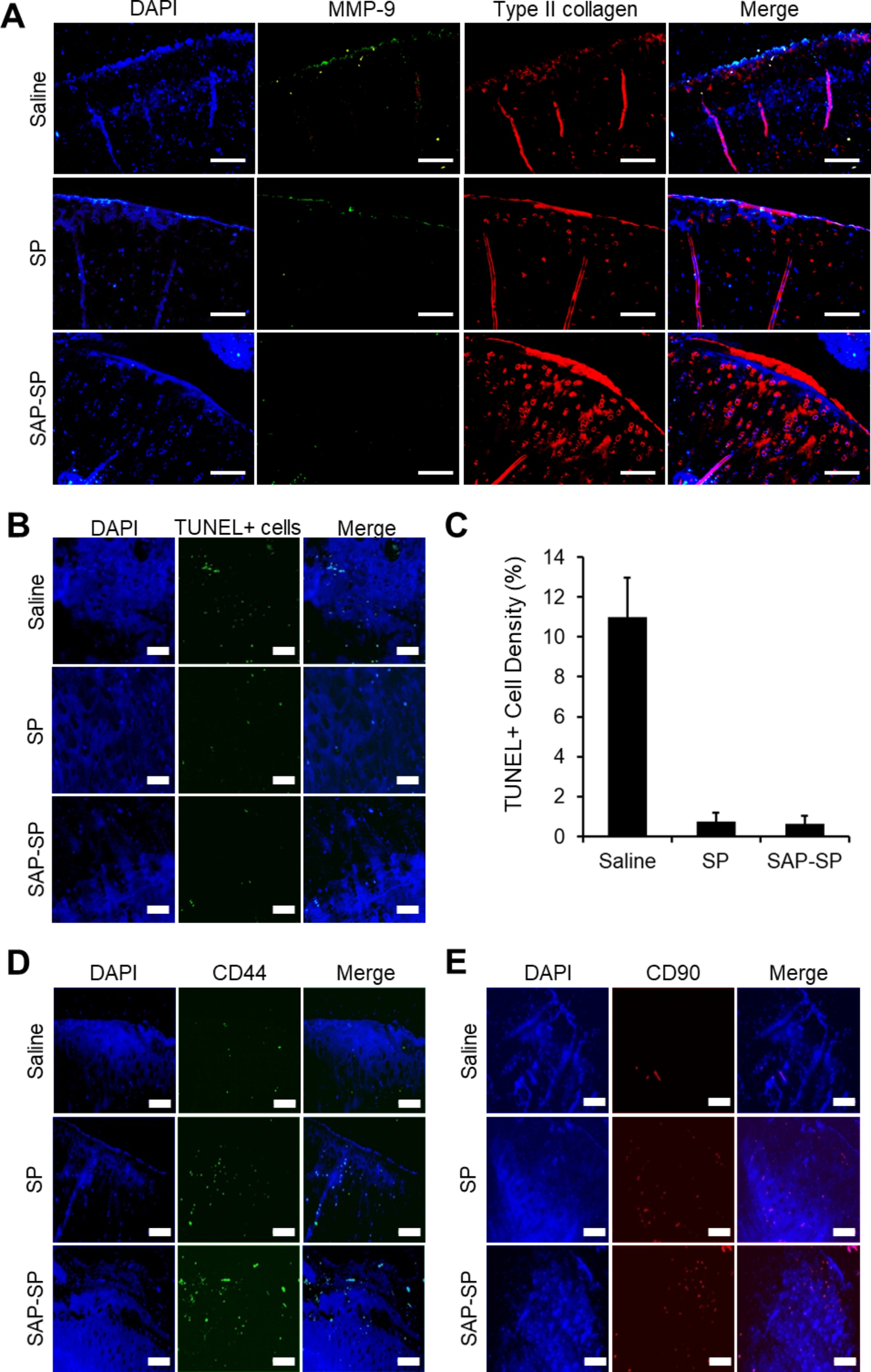
**(A)** MMP-9 and type II collagen were detected by immunofluorescence staining. (DAPI: blue, MMP-9: green, type II collagen: red) Scale bars: 200 μm. **(B)** Apoptotic cells (green) in the articular cartilage regions were detected by TUNEL staining 6 months after surgical induction of OA. Scale bars: 50 μm. **(C)** The ratio of TUNEL-positive nuclei to total nuclei was quantified for each group (expressed as %, n = 3 in each group) (*, *p* < 0.05). (D-E) MSC recruitment by SAP-SP conjugates was investigated. Representative images of articular cartilage defect sites from each group after CD44 (green) and CD90 (red) staining. Scale bars: 100 μm

Immunofluorescence staining was performed for evaluating MMP-9, which is involved in osteochondral degradation and type II collagen, a major component of cartilage (Fig. 4A). A large amount of MMP-9 was detected on the surface of the articular cartilage in the saline group, while a less amount was detected in the same area in the SP group. MMP-9 expression was nearly undetectable in the SAP-SP group. Type II collagen was almost undetectable on the articular surface in the saline group; however, a small amount was detected in the SP group, and a large amount was detected along the articular surface in the SAP-SP group. TUNEL assay was conducted for determining the effect of SAP-SP on the inhibition of degradation, regeneration of subchondral bone, and inhibition of chondrocyte apoptosis (Fig. 4B and C). Some TUNEL-positive cells were detected in the saline group (11.00 ± 1.94%), and the number of TUNEL-positive cells was reduced in the SP (0.76 ± 0.43%, *p* < 0.001) and SAP-SP (0.64 ± 0.40%, *p* < 0.001) groups.

To examine the MSC recruiting effect of SAP-SP, we stained for the MSC markers CD44 and CD 90 (Fig. 4D and E). In the saline group, the expression of CD44 and CD90 markers was relatively low, and their expression was higher in the SP group. In the SAP-SP group, CD44 + cells were more abundant than in the saline and SP groups and the number of CD90 + cells was similar to that in the SP group.

**Fig. 5:**
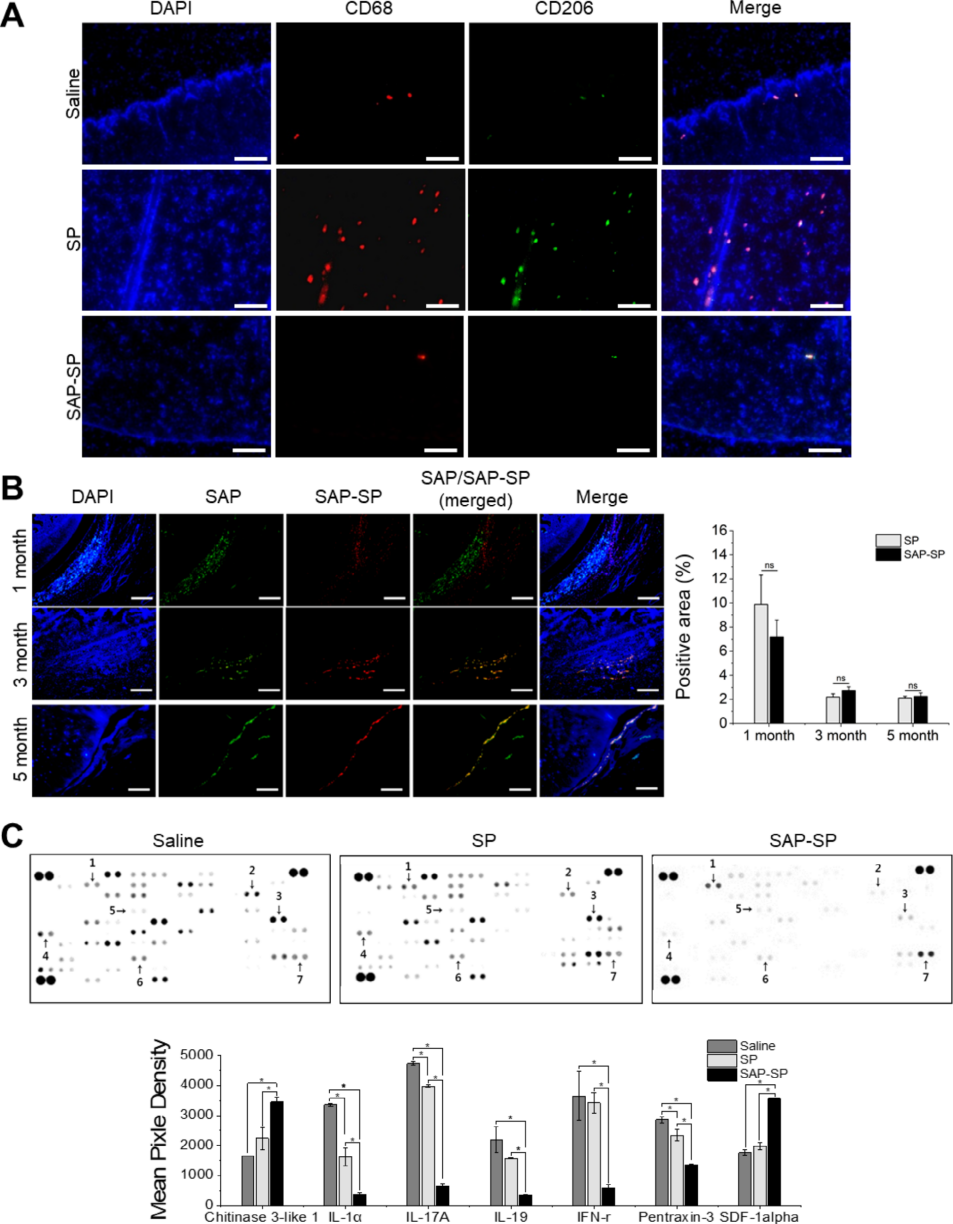
**(A)** Macrophage markers, CD68 and CD206, were stained to determine the immunogenic effect of SAP-SP (DAPI: blue, CD68: red, CD206: green). Scale bars: 200 μm. **(B)** SAP and SAP-SP were tagged with biotin and TAMRA to confirm the effect of injected SAP-SP in the articular knee joint. At 1, 3, and 5 months after injection, immunofluorescent staining was performed to analyze SAP-SP disappearance in vivo, and the positive area was quantified using images by Image J (*, p < 0.05). Scale bars: 200 μm. **(C)** Pro-inflammatory cytokines and regeneration-related cytokines were evaluated using the dot blot assay (*, *p* < 0.05)

To examine the immunogenic effect of SAP-SP on OA, we evaluated two macrophage markers, CD68 and CD206, using immunofluorescence staining (Fig. 5A). CD68 + and CD206 + cells were found on the cartilage surface and in the subchondral bone of the saline group but were concentrated on the subchondral bone area of the SP group. In the SAP-SP group, only slight staining was detected in the subchondral bone area.

To analyze the in vivo disappearance of SAP-SP, we tracked SAP and SAP-SP tagged with biotin and TAMRA. In this model, the materials were injected 1 month after arthritis induction surgery, and the experiment was terminated after 6 months. Therefore, the measurement periods were 1, 3, and 5 months, respectively. SAP and SAP-SP were present for five months; however, their amounts decreased (Fig. 5B). The positive rate was 9.7% in the SAP group and 7.2% in the SAP-SP group at 1 month but decreased to 2.1% and 2.2% at 5 months, respectively. There were no statistically significant differences in the positive areas between the two groups during the entire observation period. This seems to be because the main component of SAP-SP is SAP. Therefore, the mechanical properties that determine the stability of a hydrogel are similar to those of SAP. SAP and SAP-SP infiltrated the synovium and cartilage surface, rather than aggregating or dispersing in the joint cavity.

We extracted the synovial fluid from the joint cavity to evaluate the inflammatory status, and pro-inflammatory cytokines and regeneration-related cytokines were evaluated using a dot blot assay (Fig. 5C). The expression of regeneration-related cytokines, CHI3L1, and SDF-1α were slightly higher in the SP group and markedly higher in the SAP-SP group compared with that in the saline group. However, the levels of the pro-inflammatory cytokines IL-1α, IL-17 A, IL-19, IFN-γ, and PTX-3 were markedly lower in the SAP-SP group than in either the saline or SP group.

### The effects of SAP-SP on OA in the chemical induction OA models

Representative micro-CT 3D images of the OA guinea pig model are shown in Fig. 1B. To observe cartilage degeneration in the MIA model, we examined the knee joints with a micro-CT scanner 6 and 12 weeks after the SAP-SP injection into the knee joint cavity. In the saline group, the cartilage surfaces of the knee specimens showed severe loss and erosion of the cartilage in the medial tibial plateaus compared to the normal knee at 6 weeks. Similarly, the surfaces of the femur specimens showed erosion and destruction, which was lower in the SAP-SP-treated group compared to that in the saline group. At 12 weeks after intra-articular injection of treatment, the lesions in the saline group were more severe than at 6 weeks; however, SAP-SP-treated cartilage specimens showed a reduction in lesion severity compared with the saline group.

For observing the microscopic appearance of the articular cartilage after treatment, the cartilage specimens were stained with H&E, alcian blue, and Masson’s trichrome stain (Fig. 1C). Six weeks after each treatment, the saline-treated specimens showed non-smooth cartilage surfaces and loosely distributed chondrocytes. The SAP-SP-treated group specimens exhibited irregular and non-smooth surfaces, abnormal chondrocyte distributions, and thin cartilage layers, whereas the normal group specimens exhibited smooth surfaces and well-distributed cartilage cells and matrices. Twelve weeks after each treatment, the saline-treated group showed severe OA, with chondrocyte hypocellularity, loose chondrocyte density in the knee, and structural disorganization. However, the SAP-SP-treated group showed a better therapeutic effect, with increased chondrocyte distribution, than that of the specimens at 6 weeks. The alcian blue staining revealed that the SAP-SP-treated group specimens showed weaker accumulation of sulfated GAGs than the normal group, but the chondrocyte lacunae were round and similar to that of the normal cartilage. The saline-treated group specimens were barely stained with alcian blue. Furthermore, the SAP-SP specimens demonstrated sufficient amounts of GAGs and better distribution of GAGs in the cartilage tissues than the other groups at 12 weeks after treatment. The saline-treated group specimens displayed severe irregularity and cartilage surface layer loss at 12 weeks after treatment. Masson’s trichrome staining demonstrated sufficient accumulation of collagen in the SAP-SP-treated group and revealed the presence of regenerated cartilage. However, saline-treated samples showed partial Masson’s trichrome staining and caved cartilage surface layers. At 12 weeks after treatment, the saline-treated specimens showed more severe changes in the cartilage matrices and chondrocyte loss than at 6 weeks. Also, in the qRT-PCR results of the guinea pig chemical induction OA models, a significant decrease in TNF-α was found in the SAP-SP group compared to the non-treated group, and a significant increase in IL-10 was found in the SAP-SP group at 12 weeks compared to the non-treated group (Fig. S2). These results indicated that SAP-SP treatment was effective for cartilage regeneration in the MIA-induced OA model.

**Fig. 6:**
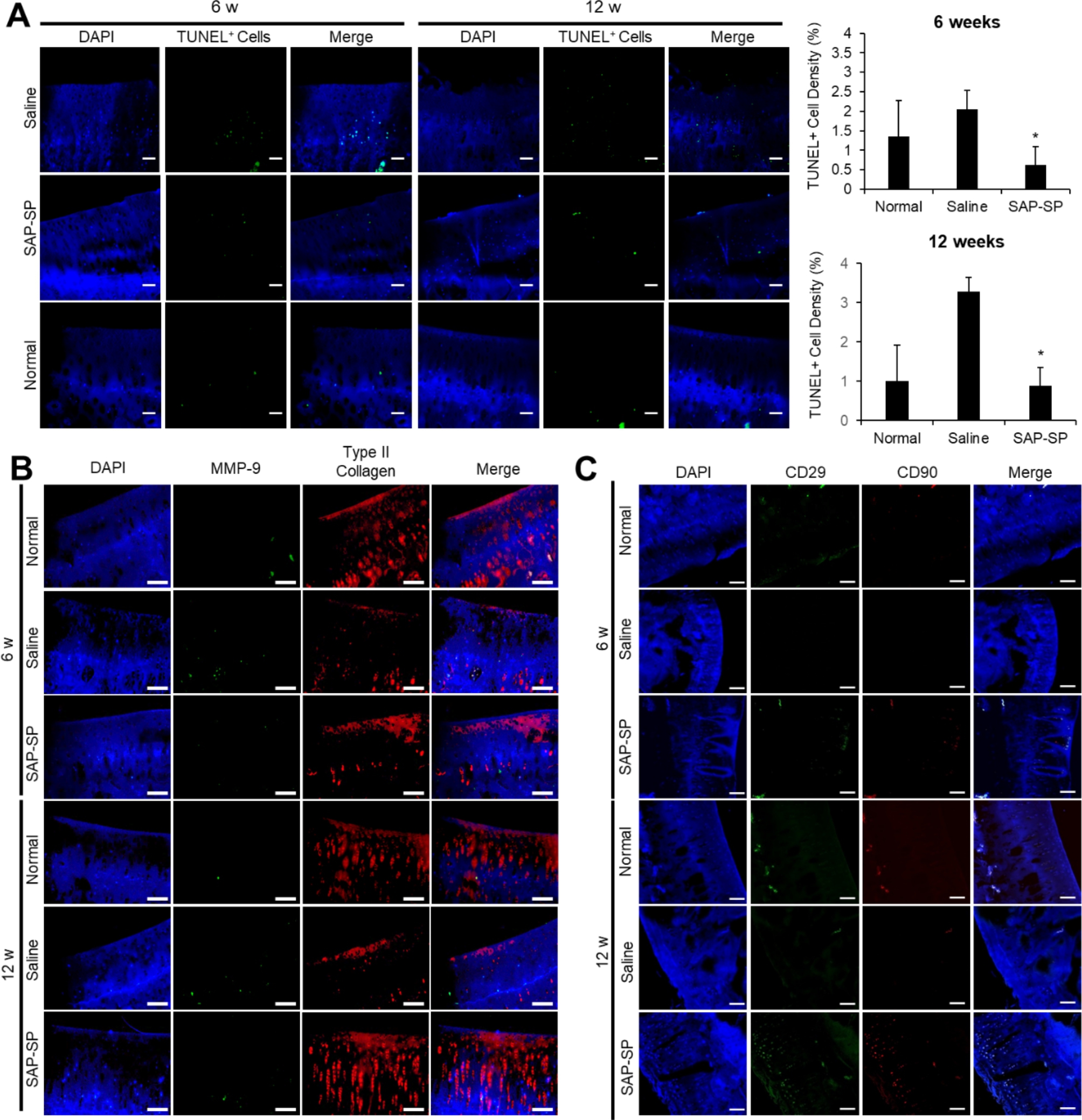
**(A)** Apoptosis in the knee joint cartilage was detected 6 and 12 weeks after chemical induction of OA by TUNEL staining in the articular cartilage regions. Apoptotic cells were stained with green positive fluorescence. The ratio of TUNEL-positive nuclei to total nuclei was quantified for each group (expressed as %, n = 8 in each group). Scale bars: 25 μm (*, p < 0.05). **(B)** MMP-9 and type II collagen were detected by immunofluorescence staining. (DAPI: blue, MMP-9: green, type II collagen: red) Scale bars: 100 μm. **(C)** Mesenchymal stem cell (MSC) recruitment by SAP-SP conjugates was investigated. SAP-SP conjugates are effective for the recruitment of MSCs into the arthritic defect region. The representative images of articular cartilage defect sites from each group after CD29 and CD90 staining. Scale bars: 100 μm

To observe the apoptotic effect of cartilage cells in the OA model, we performed a TUNEL assay 6 and 12 weeks after treatment. TUNEL positive cells were detected in large amounts in the saline-treated group specimens 6 weeks after treatment (Fig. 6A). Interestingly, the density of apoptotic cells in the SAP-SP-treated specimens was similar to that in the normal guinea pig cartilage specimens. The saline-treated group specimens showed more TUNEL-positive cells than the other groups at 12 weeks after treatment. On the basis of these results, we demonstrated the anti-apoptotic effect of SAP-SP in an OA animal model.

Immunofluorescence analysis of MMP-9 and type II collagen showed that at 6 weeks after treatment, MMP-9 was abundant in the saline group and less abundant in SAP-SP knee specimens, similar to the results in normal cartilage (Fig. 6B). At 12 weeks after treatment, MMP-9 expression in the SAP-SP-treated group was lower than that in the saline-treated group; however, the SAP-SP expression level was higher than that in the 6-week sample. Nevertheless, the SAP-SP treatment resulted in significant suppression of MMP-9 expression compared with saline treatment alone in the MIA-induced OA model. The type II collagen immunofluorescence efficacy of SAP-SP was demonstrated 6 and 12 weeks after treatment in the OA model. The SAP-SP group specimens showed partially deep expression of type II collagen 6 weeks after treatment, whereas type II collagen was evenly expressed in the articular cartilage surface of normal knees. In the saline-treated group, type II collagen was barely detected in the OA knee joint sample 6 weeks after treatment. Twelve weeks after treatment, the articular cartilage layer type II collagen was similar between the SAP-SP-treated group and normal cartilage group; lower expression of type II collagen was detected in the saline-treated group.

To determine MSC recruitment to the defect sites, we stained for CD29 and CD 90 as MSC markers for guinea pigs. In all groups except the SAP-SP group, the expression of CD29 and CD90 markers was low in rare positive cells, whereas the number of CD29 + cells and CD90 + cells were more in the SAP-SP group (Fig. 6C and Fig. S3A ).

### The effects of SAP-SP on OA in the spontaneous model

To observe cartilage regeneration from the treatment of the spontaneous model of Dunkin Hartley guinea pigs, the knee joints were examined at 44 weeks using micro-CT. The macroscopic observation of the spontaneous model showed no demonstrable change in any experimental group (Fig. 2B).

More detailed and exquisite analysis was required to examine the spontaneous model; therefore, we conducted histological analysis, including H&E, Alcian blue, and Masson’s trichrome staining (Fig. 2C). In H&E staining to identify the cartilaginous tissue structure and chondrocyte morphology, the non-treatment group (control) showed a damaged and non-smooth cartilage surface layer, as well as a decreased distribution of chondrocytes in parts of the superficial zone, compared to the other treatment groups. Alcian blue staining showed an abundant distribution of GAGs in the cartilaginous tissues. There was a significant difference between the groups treated with a single SAP-SP injection and other groups (Fig. 2C). In particular, the single SAP-SP injection group specimens showed an abundance of GAGs, indicating that repeated injection of SAP-SP may be helpful for cartilage regeneration. Furthermore, the tidemark, which is a cartilage layer structure, was clearly identifiable in the repeated SAP-SP injection group. In contrast, GAGs showed weak staining in the control group. Similarly, Masson’s trichrome staining revealed an abundant accumulation of collagen. The staining of blue-colored collagen was weaker in the control group than in the other treatment groups.

**Fig. 7:**
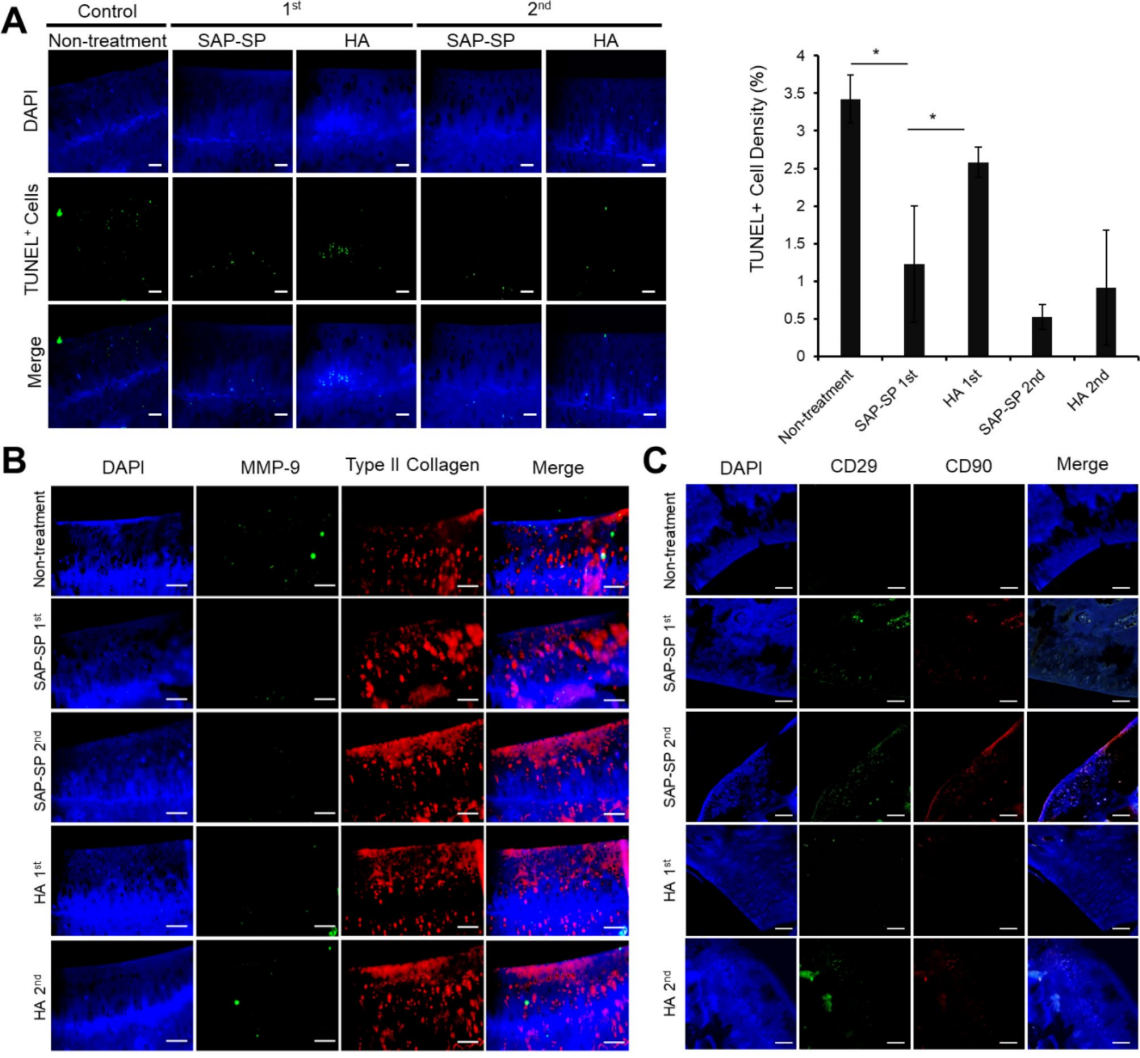
**(A)** Apoptotic cells (green) in the knee joint cartilage were detected 6 and 12 weeks after spontaneous induction of OA by TUNEL staining in the articular cartilage regions. The ratio of TUNEL-positive nuclei to total nuclei was quantified in each group (expressed as %, n = 4 in each group). Scale bars: 25 μm (*, *p* < 0.05). **(B)** MMP-9 and type II collagen were detected by immunofluorescence staining. (DAPI: blue, MMP-9: green, type II collagen: red) Scale bars: 100 μm. **(C)** MSC recruitment of SAP-SP conjugates was investigated. The representative images of articular cartilage defect site from each group after CD29 (green) and CD90 (red) staining. Scale bars: 100 μm

Immunofluorescence analysis of type II collagen showed that the repeated SAP-SP injection group showed a uniform and distinctly visible cartilage surface layer. In contrast, type II collagen was only partially expressed in all but the repeated SAP-SP injection group. Type II collagen expression was particularly low in the cartilage tissue of the control group. Furthermore, we performed a TUNEL assay to confirm the degree of apoptotic cartilage in spontaneous OA, as well as to identify the anti-apoptotic effects according to treatment regimen and frequency (Fig. 7A). In the non-treatment group, a variety of apoptotic cells were detected; fewer apoptotic cells were observed in the single SAP-SP and HA injection groups. However, the groups administered the repeated SAP-SP injection and repeated HA injection showed a greater chondrocyte anti-apoptotic effect.

Similar to the procedure followed in the MIA model, MMP and type II collagen immunostaining was performed in the spontaneous guinea pig model (Fig. 7B). Several MMP-9 markers were expressed in the non-treated group than in the SAP-SP- and HA-treated groups. These effects were probably due to the increased production of MMP-9, which is involved in tissue degradation in naturally occurring OA. In contrast, the SAP-SP-and HA-treated groups showed a lower expression of MMP-9 compared to the control group.

As in the guinea pig chemical induction OA model, MSC recruitment to the defect sites was only demonstrated in the SAP-SP group; CD29 + and CD90 + cells were observed occasionally in the SAP-SP group; however, only rare positively stained cells were observed in the HA-treated or non-treated group (Fig. 7C and Fig. S3B).

### Human synovial fluid analysis

**Fig. 8:**
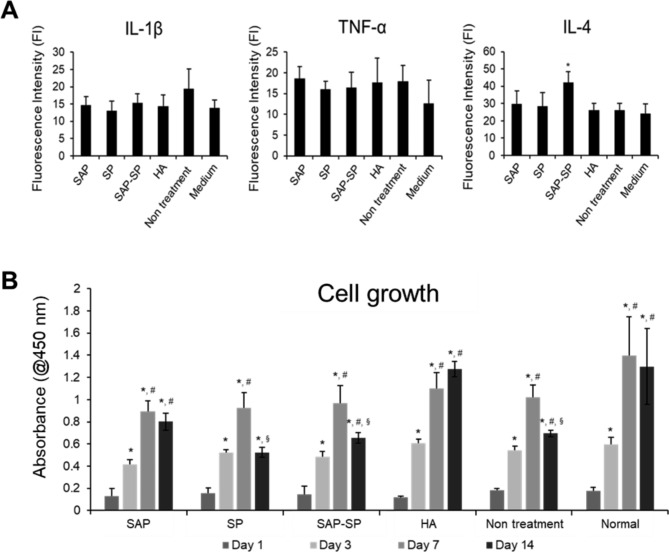
**(A)** Luminescent cytokine assays for the human synoviocytes cultured with OA synovial fluids. There was no significant difference in IL-1β and TNF-α levels between groups; however, the SAP-SP group showed a significant increase in IL-4 levels compared to all other groups (*, *p* < 0.01). **(B)** Cell growth studies for the human synoviocytes cultured with OA synovial fluids. *, #, and § indicate significant differences with Day 1, Day 3, and Day 7 respectively (p < 0.05)

We used human synoviocytes and synovial fluid from OA patients to examine the effects of SAP-SP on human samples in vitro. We examined the growth of synoviocytes and the change in secreted pro- or anti-inflammatory cytokine profiles while culturing the cells with the treatments and synovial fluids (Fig. 8). In the human synovial fluid analysis, no significant differences in IL-1β and TNF-α levels were observed between the groups; however, the SAP-SP group showed a significant increase in IL-4 levels compared to all other groups (*p* < 0.01, Fig. 8A). In the cell growth studies, all groups showed similar growth patterns, except for the HA group. Growth increased continuously until day 7 and decreased because of saturation on day 14; growth continued to increase until day 14 in the HA group (Fig. 8B).

## Discussion

In this study, we established different injection time points for each animal model according to the time taken for the full development of OA. A synovial joint fluid analysis was performed for investigating the inflammatory status of the joint cavity. Findings from in vivo studies can differ from those of in vitro analyses [45, 46]; therefore, the results of the joint fluid analysis from our in vivo study are more meaningful than previous results.

BMD is reportedly increased in OA because of subchondral bone sclerosis [47]. This abnormal increase in the BMD can cause inflammation and pain in the clinical situation. We observed that BMD decreased in the SAP-SP group because SAP-SP decreased the subchondral bone sclerosis, which suggests a positive effect on the OA. However, BMD increased in the saline, SAP, and SP groups (Fig. 3B).

The high expression of ACAN in the SAP-SP group according to our results supports SAP-mediated modulation of the SP effect (Fig. S1). Because mesenchymal progenitor cells may be involved in the intrinsic ability body to repair OA, we used CD44 and CD90 MSC markers to investigate intrinsic MSC recruitment by SP and SAP-SP [48]. CD44 expression was higher in the SAP-SP group than in the SP group, whereas CD90 expression was similar between the two groups (Fig. 4D and E). There was no clear difference in the characteristics of the two surface markers, which are representative MSC markers associated with knee OA [49]. As CD44, a hyaluronan receptor, serves as a modulator of chondrocyte metabolism [50], CD44 + MSCs recruited by SAP-SP are more important for cartilage regeneration. CD105 is also an important surface marker that promotes chondrogenesis of MSCs in humans through Smad2 signaling [51]. However, we did not investigate CD105 + surface markers in this study because we used rabbits, which do not express CD105 + surface markers [52]. In addition, we did not directly analyze the chondrogenic differentiation of MSCs because we expected an influence of MSCs on OA progression through paracrine effects after recruitment.

Apoptosis, determined using the TUNEL assay, was markedly inhibited in the SAP-SP and SP groups, but not in the SAP group, indicating that SAP did not directly affect apoptosis, possibly because preventing chondrocyte apoptosis in OA requires early inhibition of the cascade cycle [53]. Therefore, the SP and SAP-SP groups showed no significant differences in the inhibition of apoptosis (Fig. 4B). Improvement in tissue histology, shown using H&E and alcian blue staining, demonstrated that SAP-SP could serve as a therapeutic agent for treating degenerative OA. SAP-SP decrease inflammation caused by OA, enhance cartilage regeneration, and inhibit chondrocyte apoptosis. These effects were demonstrated by immunofluorescence staining, qRT-PCR, and TUNEL assays.

Macrophage markers were used for evaluating the immunogenic effects of SAP-SP injection into the joint cavity. Although macrophage infiltration in OA occurs mainly in the synovial membrane, we investigated the expression of macrophage markers in cartilage and subchondral bone. This was done because CD68 + cells are localized in the sclerotic regions of subchondral bone during OA progression [54, 55]. We used the general macrophage marker CD68 and the M2 macrophage (pro-healing/anti-inflammatory macrophage) marker CD206. In these two antibody staining, cells positive for only CD68 are considered M1 macrophages. Cells positive for both CD68 and CD206 are considered M2 macrophages and inflammation or regeneration can be confirmed. The markers were concentrated in the subchondral bone area of the SP group. These markers were barely detected and were limited to the subchondral bone area in the SAP-SP group (Fig. 5A and Fig. S4). Thus, neither SP nor SAP-SP injection evoked an immunogenic reaction in the joint cavity, suggesting that SAP-SP is a safe therapeutic candidate for OA in a clinical study. We suspect that this non-immunogenic response may result from the macrophage migration inhibitory factor (MIF) induced by the inhibition of pro-inflammatory cytokines; MIF plays an important role in inflammatory arthritis [56]. We did not investigate MIF, but it may have inhibited inflammatory cytokines and decreased the inflammation caused by OA in our study.

We injected the immunofluorescent-tagged SAP-SP complex into the joint and demonstrated that the injected SAP and SAP-SP remained in the joint cavity for at least 5 months and infiltrated along the synovium and cartilage surface (Fig. 5B). In a previous study, SAPs were shown to promote cell adhesion via ionic components [22], which can explain the infiltration of SAP and SAP-SP into the tissues. We found that the distribution patterns of SAP and SAP-SP were similar, which suggests that there might be competitive infiltration along the synovium and cartilage. The injection of SAP or SAP-SP alone would have resulted in a larger amount of infiltration along the synovium and cartilage.

The levels of pro-inflammatory cytokines were markedly decreased in the SAP-SP group, indicating the inhibition of inflammation by SAP-SP (Fig. 5C). SP also inhibited pro-inflammatory cytokines but not as much as SAP-SP. This effect may have been caused by the short residence time of SP because of its lower dose. Zhou et al. [57] demonstrated a dose-dependent effect of SP on the proliferation of tendon-derived stem cells, which support our results. TGF-β is related to cartilage regeneration. Groups arranged in increasing order of TGF- β levels are as follows: saline, SP, and SAP-SP treatments (Fig. 3C). This trend can be explained by the manner in which SAP elongated and enhanced the effect of SP, which is consistent with the findings of previous studies [20, 58].

We observed that SAP-SP showed consistent therapeutic effectiveness in various animal models. The microenvironment and cellular responses of OA in humans may differ from those in animal models; therefore, we used synoviocytes and synovial fluid from human OA patients to confirm the therapeutic effect of SAP-SP. We observed that IL-1β decreased after the addition of SAP, SAP-SP, or HA although this difference was not significantly different and IL-4 increased after the addition of SAP-SP (Fig. 8A). TNF-α did not show consistent results after the addition of SAP, SAP-SP, or HA. This was consistent with the findings that synoviocytes in OA show a high expression of IL-1β and TNF-α, which can be inhibited by anti-inflammatory agents [9, 59]. Although IL-1 is highly efficacious, it is not easy to detect, even with a high-sensitivity assay such as the existing enzyme-linked immunosorbent assay (ELISA), because it is generally present at a very low concentration (Watt et al., 2016). In addition, even if the IL-1 positive cells in the synovial membrane of OA patients are counted, the probability that the OA synovial cells are IL-1 positive is low (approximately 20%) (Farahat, Yanni, Poston, & Panayi, 1993; Vincent, 2019). Therefore, it is expected that very little IL-1b was secreted from synovial cells in this experiment. This explains the absence of a statistically significant difference. A decrease in TNF-α was not evident after SAP, SAP-SP, or HA treatment; this result might be explained by the fact that TNF-α is a less specific marker than IL-1β, considering that TNF-α levels in OA cannot be discriminated from that in fibromyalgia [60]. The fact that TNF-α is not detected in hand OA in humans or in a canine OA model offers another explanation [61].

Proliferation of synoviocytes increased until day 7 but decreased after 14 days in the SAP, SP, SAP-SP, and non-treatment groups, while proliferation of synoviocytes increased continuously without decreasing until day 14 in the HA group (Fig. 8B). This effect may be due to the action of HA in the binding of the CD44 surface antigen and the expression and activation of ezrin [62–64]; SAP and SP do not have these functions.

We used different controls for the diverse OA models because we believed that the optimal controls would differ according to each animal OA model. In the spontaneous OA model, repeated HA injection was an adequate control because it produced a condition similar to the senile human OA condition. However, in the traumatic or inflammatory OA model, there was no adequate control because HA was not used, and only pain management was applied in clinics for traumatic or inflammatory OA management.

We did not investigate quantitative measures, including fluorescence-activated cell sorting and western blotting, for some parameters. In this study, we focused on the therapeutic effects of SAP-SP in various OA models. Because the therapeutic effect of SAP-SP was confirmed, a follow-up study will be conducted for demonstrating the quantitative effect of SAP-SP on OA.

The HA injection group was used as the positive control group. HA is mainly used for OA treatment in the clinical field and for verifying the efficacy of new therapeutic agents for OA. However, because of the different modes of action of SAP-SP and HA, their efficacy cannot be compared. Moreover, as a therapeutic agent for OA, pain reduction must be considered, as well as inhibition of inflammation and delay of progression. In this study, we did not investigate pain behaviors, such as the number of rears [65, 66]. After confirming the effect of SAP-SP as a therapeutic agent for OA, we plan to evaluate its pain reduction effects before applying this possible therapeutic agent in clinical trials.

## Conclusion

In this study, we found that SAP-SP exhibited anti-inflammatory action and enhanced the recruitment of intrinsic MSCs under OA conditions. These effects inhibited bone and cartilage degradation and enhanced cartilage regeneration. These results were similar to those of our previous study in a rat model and to those of other studies on SP. This study builds on our previous study in that the effects of SP were demonstrated in diverse animal models and human samples and advances the research toward a clinical study. In this study, SAP-SP showed a positive therapeutic effect in OA without any side effects, including an immune response. This contributes to the justification of SAP-SP clinical studies.

## Electronic supplementary material

Below is the link to the electronic supplementary material.

Supplementary Material 1

Supplementary Material 2

Supplementary Material 3

Supplementary Material 4

Supplementary Material 5

## Data Availability

The datasets supporting the conclusions of this article are included within the article and its additional file.

## Abbreviations

**ACAN**: Aggrecan
**ACL**: Anterior cruciate ligament
**BMD**: Bone mineral density
**CHI3L1**: Chitinase 3-like 1 protein
**GAG**: Glycosaminoglycan
**HA**: Hyaluronic acid
**IFN-γ**: Interferon-γ
**IL-1α**: Interleukin-1α
**IL-1β**: Interleukin-1β
**IL-4**: Interleukin-4
**IL-10**: Interleukin-10
**IL-17A**: Interleukin-17 A
**IL-19**: Interleukin-19
**MIA**: Monosodium iodoacetate
**MMP-9**: Matrix metalloproteinase-9
**MSC**: Mesenchymal stem cell
**OA**: Osteoarthritis
**PBS**: Phosphate-buffered saline
**PTX-3**: Pentraxin-3
**SAP**: Self-assembled peptide
**SDF-1α**: Stromal cell-derived factor-1α
**SF**: Synovial fluid
**SP**: Substance P
**TAMRA**: Tetramethylrhodamine
**TGF-β1**: Transforming growth factor beta1
**TNF-α**: Tumor necrosis factor-α
**TUNEL**: Terminal deoxy-nucleotidyl transferase deoxyuridine triphosphate nick-end labeling
