# Supplementary material for: Self-assembled peptide-substance P hydrogels alleviate inflammation and ameliorate the cartilage regeneration in knee osteoarthritis
Source: Biomater Res. 2023 May 4;27:40. doi: 10.1186/s40824-023-00387-6 (PMC10161637; doi:10.1186/s40824-023-00387-6)
Supplement: Supplementary file 2 — Supplementary Material 2 [file 40824_2023_387_MOESM2_ESM.pdf]

Reviewer Guidance - Evaluation checklist for Life Sciences

Study Design and Statistical Criteria		Present and sufficient or N/A
Sample size	a) The exact sample size (n) for each experimental group/condition (as a number, not a range). Including information about sex ratio if relevant.<br>b) Details of a power analysis included if performed, or any other relevant considerations that determined the choice of sample size.<br>c) For n < 6, individual data values should be shown rather than summary statistics alone.	Sufficient
Sample collection	a) A description of sample collection that enables the reader to understand whether the samples represent technical or biological replicates.<br>b) An explanation of inclusion/exclusion criteria if samples or organisms were excluded from the analysis.	Present
Randomization Implementation	a) How samples/ organisms were allocated to experimental groups and processed.<br>b) Full details of the randomisation procedure used (if relevant).	Sufficient
Blinding	a) For sample assessment by human investigators, a statement on whether the investigator was blinded to group assignment and outcome assessment.<br>b) How this blinding was achieved and evaluated (if relevant).	Sufficient
Replication	a) How many times each experiment shown was replicated.<br>b) An indication of the extent of variation from experiment to experiment.	Present
Statistical methods	a) Information on the statistical methods and measures used. It should be clear whether the tests are one-sided or two-sided, whether there are adjustments for multiple comparisons, whether medians or means are being shown, whether error bars are standard deviations (SD), standard error of mean (SEM) or confidence intervals.	Present
Analytical methods	a) Have the appropriate statistical tests been used to assess significance? Do the data meet the assumptions of the tests?<br>b) Is there an estimate of variation within each group of data, and is the variance similar between groups that are being statistically compared?	Present
**Reproducibility**		
Data availability	a) Have the authors provided reasonable access to the data required for review, and described how they will make their data available at publication? If it is not, does the author's rationale for not making the data available seem reasonable?	Sufficient

	b) Have the authors complied with community-established norms of data deposition (for a list of mandated data deposition and appropriate repositories please follow the table here)?	
Code availability	a) Is the code available in a public repository (or if not yet available, is it clear how it will be made available upon publication)? Is the code in a form that can be used and understood by others, including being readable at a line-by-line level in terms of syntax and comments?<br>b) Is there a clear, documented workflow (including data preparation/cleaning steps and analyses) to reproduce the results? Are all key results (figures and tables) supported by the documented workflow?	N/A
Reproducibility	a) Does the description of the methodology give potential for reproducibility of the work?	Sufficient

For further information on a specific journal, please refer to the journal's website or contact the journal's Editorial Office.

Detailed checklists are available for a number of study designs, including:

- Randomized controlled trials (CONSORT) and protocols (SPIRIT)
- Systematic reviews and meta-analyses* (PRISMA) and protocols (PRISMA-P)
- Observational studies (STROBE)
- Case reports (CARE)
- Qualitative research (COREQ)
- Diagnostic/prognostic studies (STARD and TRIPOD)
- Economic evaluations (CHEERS)
- Pre-clinical animal studies (ARRIVE)
- SAMPL guidelines
