# Supplementary material for: Self-assembled peptide-substance P hydrogels alleviate inflammation and ameliorate the cartilage regeneration in knee osteoarthritis
Source: Biomater Res. 2023 May 4;27:40. doi: 10.1186/s40824-023-00387-6 (PMC10161637; doi:10.1186/s40824-023-00387-6)
Supplement: Supplementary file 3 — Supplementary Material 3 [file 40824_2023_387_MOESM3_ESM.docx]

ACAN: Aggrecan

ACL: Anterior cruciate ligament

BMD: Bone mineral density

CHI3L1: Chitinase 3-like 1 protein

GAG: Glycosaminoglycan

HA: Hyaluronic acid

IFN-γ: Interferon-γ

IL-1α: Interleukin-1α

IL-1β: Interleukin-1β

IL-4: Interleukin-4

IL-10: Interleukin-10

IL-17A: Interleukin-17A

IL-19: Interleukin-19

MIA: Monosodium iodoacetate

MMP-9: Matrix metalloproteinase-9

MSC: Mesenchymal stem cell

OA: Osteoarthritis

PBS: Phosphate-buffered saline

PTX-3: Pentraxin-3

SAP: Self-assembled peptide

SDF-1α: Stromal cell-derived factor-1α

SF: Synovial fluid

SP: Substance P

TAMRA: Tetramethylrhodamine

TGF-β1: Transforming growth factor beta1

TNF-α: Tumor necrosis factor-α

TUNEL: Terminal deoxy¬nucleotidyl transferase deoxyuridine triphosphate nick-end labeling
