# Supplementary material for: Self-assembled peptide-substance P hydrogels alleviate inflammation and ameliorate the cartilage regeneration in knee osteoarthritis
Source: Biomater Res. 2023 May 4;27:40. doi: 10.1186/s40824-023-00387-6 (PMC10161637; doi:10.1186/s40824-023-00387-6)
Supplement: Supplementary file 4 — Supplementary Material 4 [file 40824_2023_387_MOESM4_ESM.docx]

Supplementary Information

**Self-assembled peptide-substance P hydrogels alleviate inflammation and ameliorate the cartilage regeneration in knee osteoarthritis**

Sang Jun Kim^a,b,1^, Ji Eun Kim^c,d,1^, Goeun Choe^c,1^, Da Hyun Song^a^, Sun Jeong Kim^e,f^, Tae Hee Kim^c^, Jin Yoo^c^, Soo Hyun Kim^c,d^, Youngmee Jung^c,e,*^

^a^Department of Physical and Rehabilitation Medicine, Seoul Jun Rehabilitation Clinic and Research Center, Seoul, Republic of Korea

^b^Stem Cell & Regenerative Medicine Institute, Samsung Medical Center, Seoul, Republic of Korea

^c^Center for Biomaterials, Biomedical Research Institute, Korea Institute of Science and Technology (KIST), Seoul, Republic of Korea

^d^KU-KIST Graduate School of Converging Science and Technology, Korea University, Seoul, Republic of Korea

^e^Stem Cell Institute, ENCell Co. Ltd, Seoul, Republic of Korea

^f^Cell and Gene Therapy Institute, Samsung Medical Center, Seoul, Republic of Korea

^g^School of Electrical and Electronic Engineering, YU-KIST Institute, Yonsei University, Seoul, Republic of Korea

^1^ These authors contributed equally to this work.

* Corresponding author. Email: winnie97@kist.re.kr

**Contents:**

**Figure S1**. In the qRT-PCR results of the rabbit surgical induction OA models, a significant increase in TGF-β1 was found in the SP and SAP-SP groups compared to the saline group, and the difference in TGF-β1 between the SP and SAP-SP groups was also significant (*, p < 0.05 by Kruskal–Wallis and post-hoc Mann–Whitney U tests with Bonferroni correction). The gene expression was normalized to a housekeeping gene, GAPDH.

**Figure S2**. In the qRT-PCR results of the guinea pig chemical induction OA models, a significant decrease in TNF-α was found in the SAP-SP group compared to the non-treated group, and a significant increase in IL-10 was found in the SAP-SP group at 12 weeks compared to the non-treated group. The gene expression was normalized to a house keeping gene, GAPDH.

**Figure S3.** High-resolution image of MSC recruitment by SAP-SP conjugates in (A) chemical induction OA model and (B) spontaneous model. Representative images of articular cartilage defect sites from each group after CD29 and CD90 staining. Scale bars: 50 μm.

**Figure S4**. Low-resolution images of the macrophage markers (CD68 and CD206) immunostaining images to determine the immunogenic effect of SAP-SP in the surgical OA model. Scale bars: 100 μm.

**
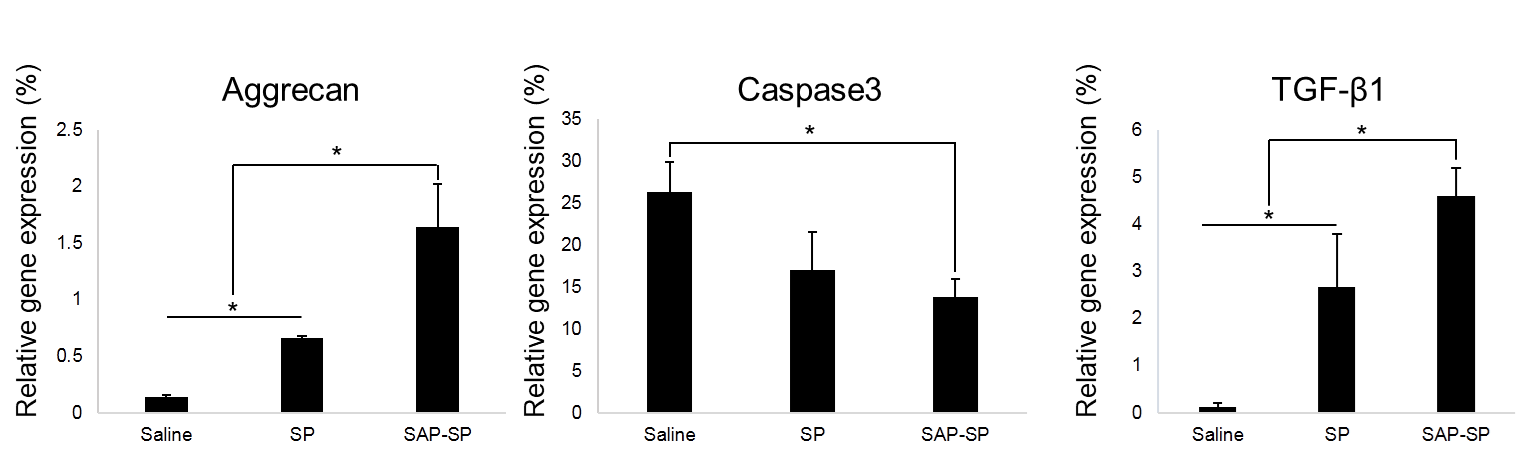
**

**Figure S1**. In the qRT-PCR results of the rabbit surgical induction OA models, a significant increase in TGF-β1 was found in the SP and SAP-SP groups compared to the saline group, and the difference in TGF-β1 between the SP and SAP-SP groups was also significant (*, p < 0.05 by Kruskal–Wallis and post-hoc Mann–Whitney U tests with Bonferroni correction). The gene expression was normalized to a housekeeping gene, GAPDH.

**
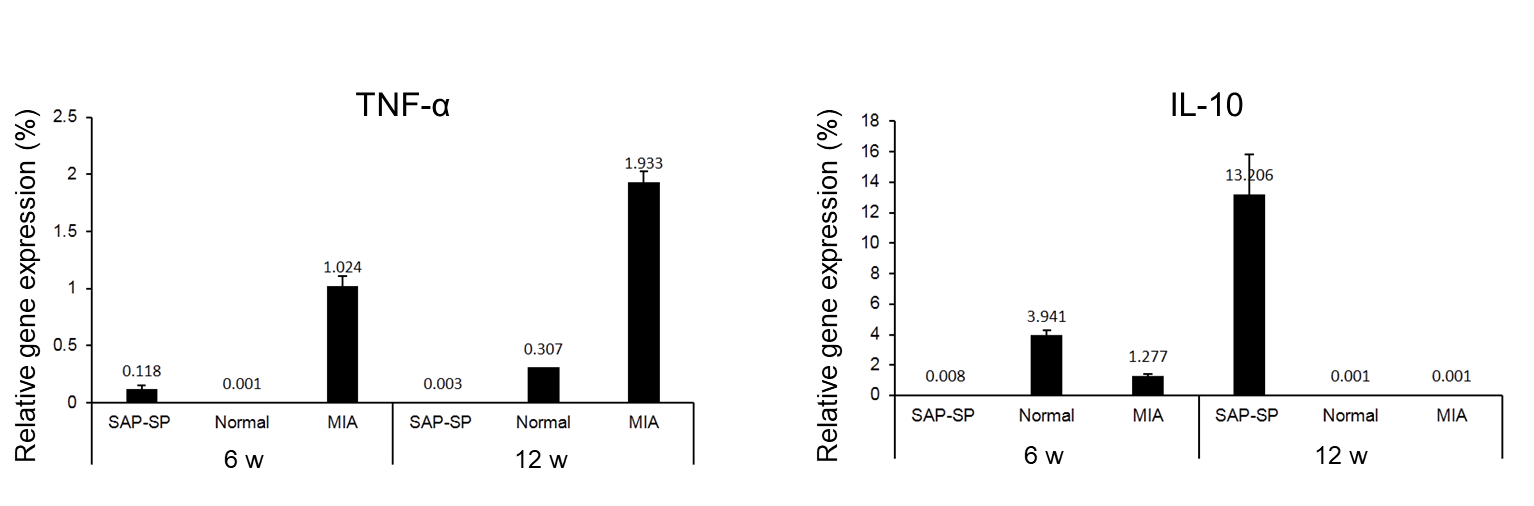
Figure S2**. In the qRT-PCR results of the guinea pig chemical induction OA models, a significant decrease in TNF-α was found in the SAP-SP group compared to the non-treated group, and a significant increase in IL-10 was found in the SAP-SP group at 12 weeks compared to the non-treated group. The gene expression was normalized to a housekeeping gene, GAPDH.


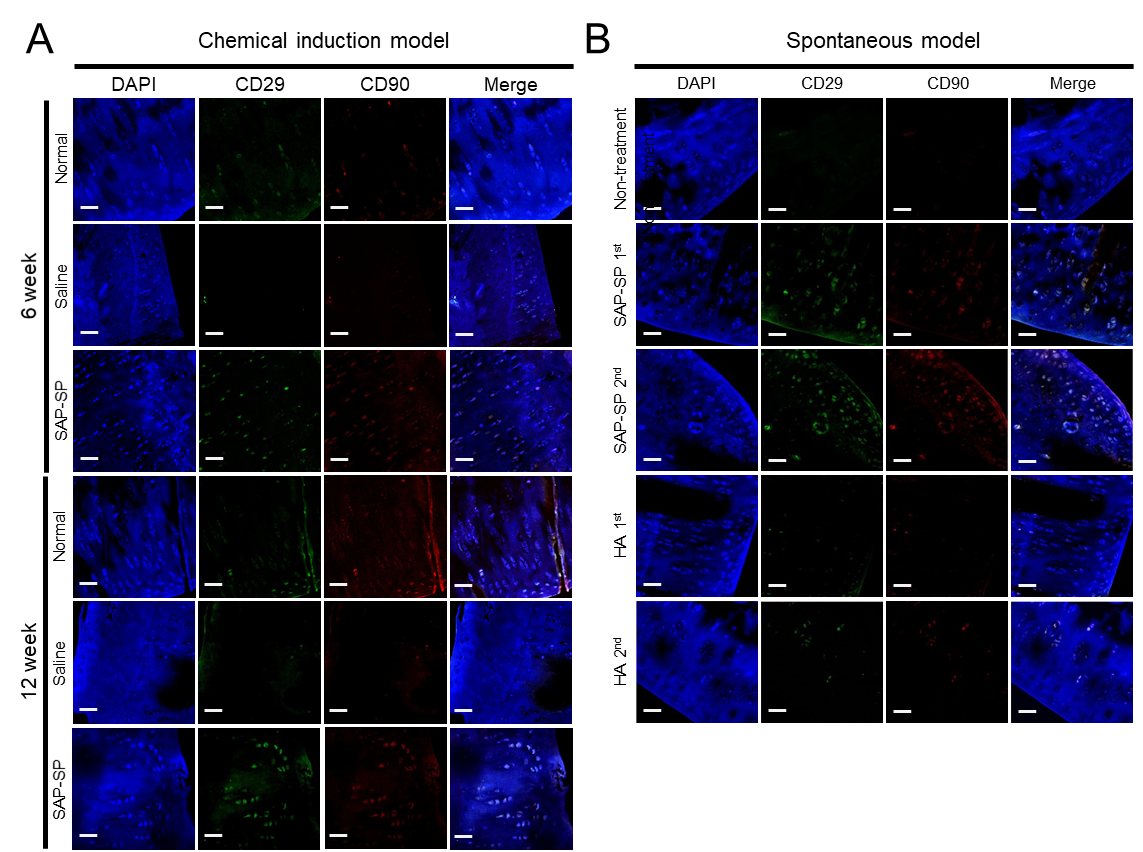


**Figure S3.** High-resolution image of MSC recruitment by SAP-SP conjugates in (A) chemical induction OA model and (B) spontaneous model. Representative images of articular cartilage defect sites from each group after CD29 and CD90 staining. Scale bars: 50 μm.


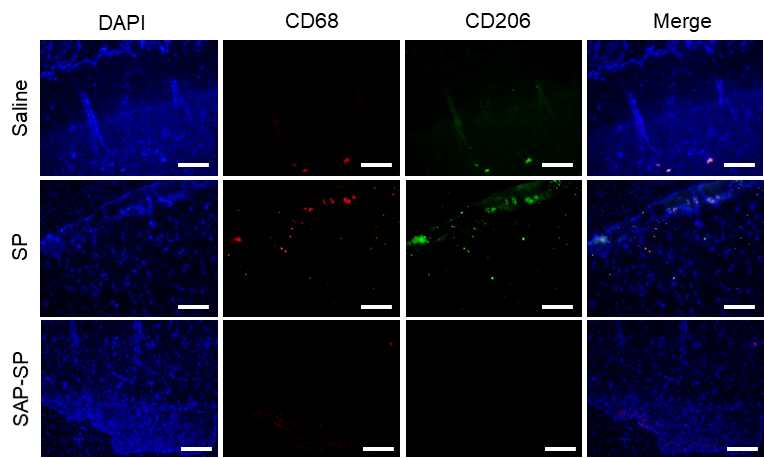


**Figure S4**. Low-resolution images of the macrophage markers (CD68 and CD206) immunostaining images to determine the immunogenic effect of SAP-SP in the surgical OA model. Scale bars: 100 μm.
