# Supplementary material for: Self-assembled peptide-substance P hydrogels alleviate inflammation and ameliorate the cartilage regeneration in knee osteoarthritis
Source: Biomater Res. 2023 May 4;27:40. doi: 10.1186/s40824-023-00387-6 (PMC10161637; doi:10.1186/s40824-023-00387-6)
Supplement: Supplementary file 1 — Supplementary Material 1 [file 40824_2023_387_MOESM1_ESM.docx]

Center for Biomaterials, Korea Institute of Science and Technology

Hwarangro 14-gil 5, Seoungbuk-gu, Seoul 136-791, Korea

Apr 18, 2023

Dear Professor Byeongmoon Jeong,

Editors-in-Chief in Biomaterials Research

**Dear Editor:**

I wish to submit the revised article [BMRS-D-23-00084] paper for publication in Biomaterials Research., titled “Self-assembled peptide-substance P hydrogels alleviate the inflammation and ameliorate the cartilage regeneration in knee osteoarthritis.” The paper was co-authored by Sang Jun Kim, Ji Eun Kim, Goeun Choe, Da Hyun Song, Sun Jeong Kim, Tae Hee Kim, Jin Yoo, Soo Hyun Kim.

We appreciate the opportunity to revise the manuscript and we are grateful for the valuable suggestions made by the reviewers. We have revised the manuscript to address the reviewers’ comments by performing additional discussion and corrections. We believe that the reviewers’ inputs have further enhanced the quality of our manuscript. Here, we provide point-by-point responses. As well, changes made in the paper are presented here in yellow highlights.

We are much obliged for your time and decision.

Youngmee Jung, Ph.D.

Principal Research Scientist/Adjunct Professor

Center for Biomaterials, Korea Institute of Science and Technology (KIST)

School of Electrical and Electronic Engineering, Yonsei University

5 Hwarangro 14-gil, Seongbuk-gu, Seoul, 02792, Republic of Korea

Telephone: +82-2-958-5348

Fax: +82-2-958-5308

E-mail: winnie97@kist.re.kr
